# Genetic tagging of active neurons in auditory cortex reveals maternal plasticity of coding ultrasonic vocalizations

**DOI:** 10.1038/s41467-018-03183-2

**Published:** 2018-02-28

**Authors:** Gen-ichi Tasaka, Casey J. Guenthner, Amos Shalev, Omri Gilday, Liqun Luo, Adi Mizrahi

**Affiliations:** 1 Department of Neurobiology, The Hebrew University of Jerusalem, Jerusalem, 91904 Israel; 2 The Edmond and Lily Safra Center for Brain Sciences, The Hebrew University of Jerusalem, Jerusalem, 91904 Israel; 3 Howard Hughes Medical Institute, Stanford University, Stanford, CA 94305 USA; 4 Department of Biology, Stanford University, Stanford, CA 94305 USA

## Abstract

Cortical neurons are often functionally heterogeneous even for molecularly defined subtypes. In sensory cortices, physiological responses to natural stimuli can be sparse and vary widely even for neighboring neurons. It is thus difficult to parse out circuits that encode specific stimuli for further experimentation. Here, we report the development of a Cre-reporter mouse that allows recombination for cellular labeling and genetic manipulation, and use it with an activity-dependent *Fos*-CreER^T2^ driver to identify functionally active circuits in the auditory cortex. In vivo targeted patch recordings validate our method for neurons responding to physiologically relevant natural sounds such as pup wriggling calls and ultrasonic vocalizations (USVs). Using this system to investigate cortical responses in postpartum mothers, we find a transient recruitment of neurons highly responsive to USVs. This subpopulation of neurons has distinct physiological properties that improve the coding efficiency for pup USV calls, implicating it as a unique signature in parental plasticity.

## Abstract (web summary)

Fos-CreER knock-in mice enable tagging of neurons that are activated within a distinct time window. Here, the authors develop a Cre reporter mouse with low baseline activation and use it to reveal the specific coding properties of auditory cortex neurons that are activated by pup calls in both naive mice and mothers.

## Introduction

The primary auditory cortex (A1) is the first cortical station encoding sound information. Unlike at the periphery (e.g., the cochlear nucleus), which primarily deconstructs sounds based on their spectral content, neuronal responses in A1, which is only a few synapses away, are often idiosyncratic. A1 responses are sparse, highly sensitive to modulation in time (amplitude modulation, frequency modulation, harmonics, context) and their coding principles are not fully understood^1^. Concurrently, it has been shown that A1 neurons encode features of auditory objects that are common in natural sounds^2^. A major challenge in auditory neuroscience is to understand cortical responses to different natural sounds, which are not perceived nor processed as a linear sum of pure tones^3,4^.

Many animals across the animal kingdom (e.g., insects, birds, whales, bats, rodents and primates) use vocalizations to communicate with other individuals in their natural environment. Here, we focus our study on cortical coding and plasticity of mouse vocalizations, which have complex spectro-temporal structure^5^. Studying these complex sounds may provide insight into cortical coding of high-dimensional sound features^6^. Specifically, we focus on newborn pup vocalizations to track a natural form of plasticity during motherhood when these sounds bear special behavioral relevance^7,8^.

Upon becoming mothers, female mice show an array of maternal behaviors, all of which are geared towards the caring of pups. In rodents, specific sets of sounds emitted by pups elicit specific maternal behaviors^9^. Two such sounds are wriggling calls (WCs) and ultrasonic vocalizations (USVs). Pup USVs are emitted as cries of help when pups are out of the nest and WCs are emitted while pups struggle to reach the mother’s nipples during suckling. Several plastic changes have been described in A1 neurons of mothers, particularly in neurons responding to USVs. These include, but are not limited to, enhanced and more reliable single neuron responses to USVs^10–13^. However, the emergent circuit properties within the mother’s auditory cortex and the brain as a whole during the postpartum period remain largely unknown^14^.

Traditionally, studying cortical representation of sounds involves blind electrophysiological recording^15,16^ or, more recently, Ca^2+^ imaging^17,18^. Notably, even when cells are targeted for recording by “cell type” markers defined by Cre lines, the neurons recorded can still be highly heterogeneous^19,20^. We took a new approach to study neurons encoding natural sounds in the auditory cortex, in which we isolate active neurons based on the expression of the immediate early gene (IEG) *Fos*^21^. Different *Fos*-based methods have been developed to tag active neurons using transgenic or knock-in mice^22–24^. We recently developed a *Fos*-CreER^T2^ knock-in mouse^25^, which utilizes the promoter of *Fos* to drive the transcription of CreER in activated neurons. This TRAP (Targeted Recombination in Active Populations) driver mouse allows temporally controlled recombination in active neurons within ~6 h around the time of a 4-hydroxytamoxifen (4-OHT) injection, which causes nuclear translocation of CreER, leading to the permanent expression of a Cre reporter. While exhibiting several advantages over other methods, TRAP may have significant levels of stochastic baseline noise (false positives). Indeed, despite the extensive use of IEG-based methods, the direct relationship between IEG expression and distinct firing properties of neurons has only rarely been tested^26^. Taking advantage of the ability of TRAP to tag and follow live neurons after recombination, we directly assessed its efficiency by recording the physiological response profiles of the recombined neurons in a complex neural network, in vivo. Additionally, we describe a new Cre-reporter mouse strain for general use and optimize its performance with the TRAP driver for targeting active neurons in the auditory cortex. We then exploit these methods to reveal coding properties of cortical neurons that are particularly responsive to natural sounds in both naive mice and following maternal plasticity.

## Results

### Optimizing TRAP with a new reporter mouse strain

**Fig. 1:**
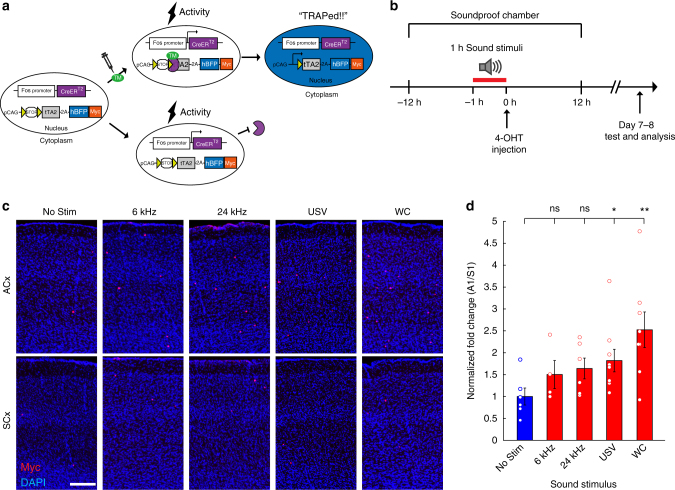
A binary expression system to TRAP active neurons in A1 with natural sounds. **a** Schematic of the *TRAPxTB* system. Left: the double transgenic mouse expresses CreER^T2^ from the *Fos* promoter, and a second cassette allows expression of two effector genes, a histone-BFP-myc nuclear marker and a tetracycline-dependent transactivator (tTA2) in a Cre-dependent manner. Bottom: in the presence of activity but without tamoxifen (TM), CreER^T2^ is expressed but retained in the cytoplasm, and no recombination can occur. Top: in the presence of activity and TM, CreER^T2^ can enter the nucleus and induce recombination and expression of histone-BFP-Myc and tTA2 (right, “TRAPed”). **b** Experimental protocol for TRAPing in A1. *TRAPxTB* mice were placed in a soundproof chamber, and were given an injection of 15 mg/kg 4-OHT after 1 h sound stimulation. After 7–8 days, mice were used for experiments. **c** Representative photomicrographs from the primary auditory (ACx, top rows) and somatosensory (SCx, bottom rows) cortices of mice stimulated with different sounds (indicated above images) and stained for myc. Scale bar, 200 μm. **d** Quantification of the density of TRAPed cells in A1 relative to their density in S1 and normalized to the ‘No Stim’ condition (mean ± SEM; ‘No Stim’ *N* = 6 mice, ‘6 kHz’ *N* = 4 mice; ‘24 kHz’ *N* = 6 mice; ‘USV’ *N* = 9 mice; ‘WC’ *N* = 8 mice). USVs and WCs but not pure tone stimuli (6 kHz and 24 kHz) induced a significant increase in the number of the TRAPed neurons in A1 (*p < 0.05; **p < 0.01; ns, not significant, post hoc Fisher’s LSD test after Kruskal–Wallis test)

Genetic targeting is often achieved through separate driver (e.g., Cre recombinase expressed in a specific pattern) and reporter systems (e.g., a Cre reporter), the combination of which determines its selectivity and efficiency. We designed a new Cre reporter mouse based on site-specific transgene integration into the *H11* locus^27^, which supports ubiquitous high-level reporter expression when combined with the *CAG* promoter^28^. The transgene allows the simultaneous expression of tTA2 and a nuclear fluorescent protein (histone2B-BFP-myc) after the excision of a transcriptional stop signal flanked by two *loxP* sites (Fig. 1a, Supplementary Fig. 1). The new reporter mouse, which we called herein “*TB*” (for tTA2-BFP), has lower noise levels as a TRAP reporter compared to common reporters like *Ai14*^29^, but similar levels of induction efficiency in cortex (Supplementary Fig. 2). Once CreER-mediated recombination occurs in target neurons, these neurons become amenable to conditional expression of any transgenes under the control of the *TRE* promoter (Supplementary Fig. 1, see below). The nuclear reporter also makes it easier to identify labeled cells, especially in vivo, without the labeling of neuronal processes associated with cytoplasmic reporters. We crossed *FosTRAP* mice to *TB* mice (hence *TRAPxTB*) and evaluated the efficiency and specificity of neuronal labeling in these mice by anatomy and physiology, focusing on A1.

To enable efficient recombination in the auditory system, we placed female mice in their home cage within a soundproof chamber for >12 h before playing sound stimuli known to induce high spiking activity in A1^20^ (Fig. 1b). To calibrate the system, we played sound for 1 h, after which we injected mice with different doses of 4-OHT and analyzed them 7–8 days later (15, 25, 50 mg/kg; Supplementary Fig. 3). Control mice went through the exact same protocol without the 1 h sound stimulation. Using this calibration, we determined the relative efficiency of different 4-OHT doses (Supplementary Fig. 3a–c) and their absolute efficiency compared to Fos staining (Supplementary Fig. 3d–f), and chose 15 mg/kg in subsequent experiments.

We first examined the cortex histologically in mice that were stimulated with different pure tone stimuli (6 kHz: *N* = 4 mice; 24 kHz: *N *= 6 mice), natural sound stimuli (USVs: *N* = 9 mice; WCs: *N* = 8 mice) or no stimuli as controls (No Stim:* N* = 6 mice). In all mice, we counted the number of TRAPed neurons in A1 as compared to the primary somatosensory cortex (S1) as an in-animal control (Fig. 1c, Supplementary Fig. 4). Mice stimulated with pure tones (either 6 or 24 kHz) showed only a small but statistically insignificant increase in the number of TRAPed neurons in A1 (Fig. 1c, d). Sound stimulation with USVs and WCs induced significantly higher levels of recombination than in controls (USV: ~1.8-fold; WC: ~2.5-fold; Fig. 1c, d). These data demonstrate that TRAPxTB can be used to genetically access subsets of neurons in A1 based on *Fos* expression in response to natural sound stimulation.

One advantage of TRAPxTB over other, more anatomically restricted methods is that neurons activated by an experience or a behavioral episode in the entire brain can be TRAPed. Indeed, the numbers of TRAPed neurons in mice exposed to WCs and to 15 mg/kg 4-OHT showed stimulus-dependent increase in additional (but not all) auditory areas like the ventral cochlear nucleus (VCN) and inferior colliculus (IC) (Supplementary Fig. 5a, b). A second advantage of the TRAPxTB system is the ability to selectively express effector genes in TRAPed neurons from transgenes under the control of the *TRE* promoter. As a proof of concept for this advantage, after TRAPing we injected into A1 adeno-associated virus (AAV) encoding either green fluorescent protein (GFP), ChETA or proteins used for rabies tracing of local circuits^30^ (Supplementary Fig. 5c–e). By calibrating the titer and serotype of each AAV we could achieve strong transgene expression largely restricted to a subset of TB+ neurons (Supplementary Fig. 5c-e). These data show that expression of additional effector transgenes can be used for further investigation of the local ensembles of TRAPed neurons.

### Electrophysiological validation of TRAPxTB in the auditory cortex

**Fig. 2:**
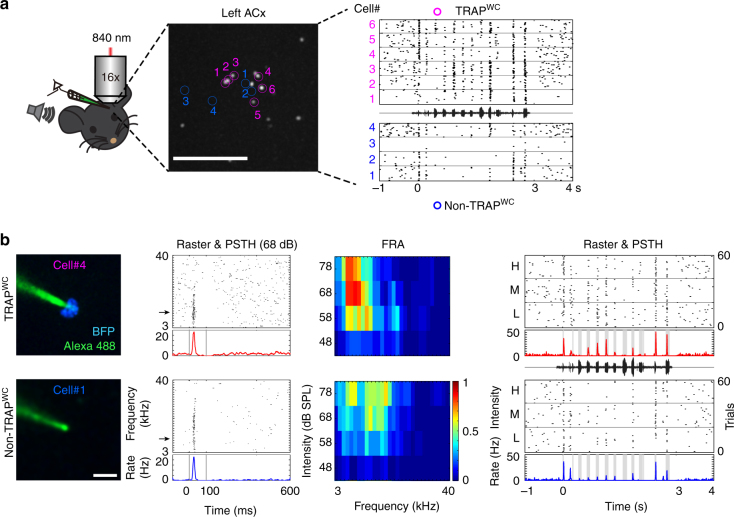
In vivo electrophysiological characterization of genetic TRAPing. **a** Left: Schematic representation of the experimental set up for in vivo two-photon targeted recording. Middle: A representative two-photon micrograph of BFP-positive neurons from the left auditory cortex (projection of 100–400 μm under the surface of A1). Scale bar 200 μm. The 6 recorded TRAPed cells and 4 non-TRAPed cells are indicated in magenta and blue, respectively. Right: Raster plots of these 10 neurons in response to WC stimuli. **b** Left, representative two-photon micrographs from two neurons whose numbers correspond to the location shown in (**a**) (Green (Alexa488), electrode; Blue (BFP), TRAPed neuron). Scale bar, 10 μm. For each neuron we recorded its responses to pure tones at 4 intensities (middle panels) and to WCs at 3 intensities (right). Rasters in response to pure tones are shown at 68 dB SPL only. Arrows in frequency axis indicate best frequency (BF) in each cell. Frequency response area (FRA) shows mean responses across all frequencies and attenuations. Color bar indicates normalized spike count. WC responses are shown at high, medium and low intensities (H, M, L; 20 trials per intensity)

To evaluate the correlation between genetic tagging and physiological response profiles in the TRAPxTB system we performed in vivo two-photon targeted loose patch recordings from animals that were stimulated with WCs. Under two-photon guidance (in ketamine-anesthetized mice) we targeted, serially, BFP-positive neurons (TRAPed) and neighboring BFP-negative neurons (non-TRAPed) as in-animal controls. All recordings were from neurons in the upper cortical layers, L2/3. An example from one representative experiment, where we recorded from a total of 10 neurons, shows that the TRAPed neurons indeed had higher spiking responses to WCs as compared to their immediate neighbors (Fig. 2a). To evaluate a more complete physiological profile of the neurons, we also recorded their spiking response to pure tones. Frequently, and in neighboring neurons, the TRAPed neuron (but not the neighbor) had strong responses to the WC despite both having very similar response profiles to the pure tones (Fig. 2b). This example suggests that the TRAPed neurons faithfully represent neurons that are strongly activated by the specific sound played during the drug-active period.

**Fig. 3:**
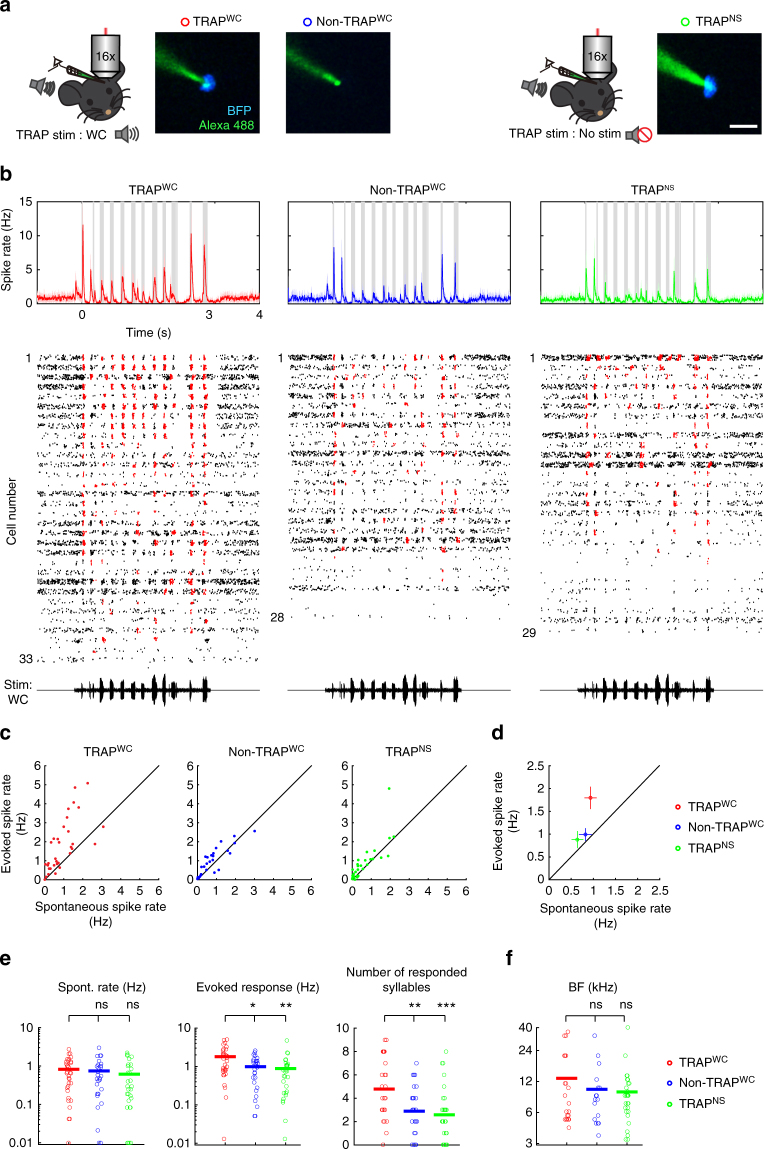
TRAPed cells over-represent subsets of highly active neurons to the inducing stimulus. **a** Schematic representation of the three neuronal groups from two experimental groups. We recorded from TRAPed and non-TRAPed neurons in mice stimulated with WC (TRAP^WC^ and non-TRAP^WC^, respectively) and from TRAPed neurons in mice that were not stimulated with sound (TRAP^NS^, green). Example photomicrograph of the electrode (green) and BFP signal (blue) is shown from each group. Scale bar, 10 μm. **b** Top: average PSTHs of all neurons in response to WC from three neuronal groups. Bottom: raster plots of all the recorded neurons (TRAP^WC^; *n* = 33 neurons, *N* = 8 mice, non-TRAP^WC^; *n* = 28 neurons, *N* = 8 mice, TRAP^NS^; *n* = 29 neurons, *N* = 9 mice). Each raster of each neuron is composed of 60 trials. Red ticks correspond to spikes that were statistically above the baseline rate around a syllable. The voltage trace of the WC stimulus is shown beneath the rasters. **c** Plots of evoked vs spontaneous spike rates from all the recorded neurons in all groups shown in (**b**). Each dot indicates the mean firing rate of a single neuron. **d** Plots of the mean ( ± SEM) evoked vs spontaneous spike rates of the neurons shown in (**b**) and (**c**). **e** Basic response properties to WC of all neurons from the three groups. Each circle represents an individual cell. The line indicates the mean. Spontaneous rate was not different between groups (*p* = 0.29, Kruskal–Wallis test). TRAPed neurons in the TRAP^WC^ mice have higher evoked spike rate and responded to a higher number of syllables in the call as compared to the other groups (*p < 0.05; **p < 0.01; ***p < 0.001; ns, not significant, post hoc Fisher’s LSD test after significant Kruskal–Wallis test). **f** Best frequencies (BFs) were not significantly different among the three groups (*p* = 0.89, Kruskal–Wallis test)

To quantitatively and systematically evaluate the physiological specificity of our system, we patched neurons from three different experimental groups: (1) TRAPed neurons in WC-stimulated mice (TRAP^WC^; *n* = 33 neurons, *N* = 8 mice), (2) non-TRAPed neighboring neurons in the same stimulated mice (non-TRAP^WC^; *n* = 28, *N* = 8) and (3) TRAPed neurons in non-stimulated mice (TRAP^NS^; *n* = 29, *N* = 9) (Fig. 3a). Spontaneous firing rates, response latencies and best frequency (BF) profiles of the experimental (TRAP^WC^) versus both control groups (non-TRAP^WC^ and TRAP^NS^) were not significantly different (Supplementary Fig. 6, see one exception therein). Responses of TRAPed neurons to the WCs were stronger as compared to both control groups (Fig. 3b–f; see the complete breakdown of the responses and statistics for pure tones and WCs in Supplementary Tables 1–4). Notably, similar recordings of the TRAPed neurons in pure tone stimulated mice did not show such specificity: no effect in the 6 kHz stimulated mice and weak effects (only for BF) in the 24 kHz stimulated mice (Supplementary Table 5). These results validate that TRAPxTB allows genetic access to a pool of cortical neurons that are indeed activated more robustly (almost twice as much) by natural sounds and not pure tones.

### Recruitment of USV-responding neurons in mothers

**Fig. 4:**
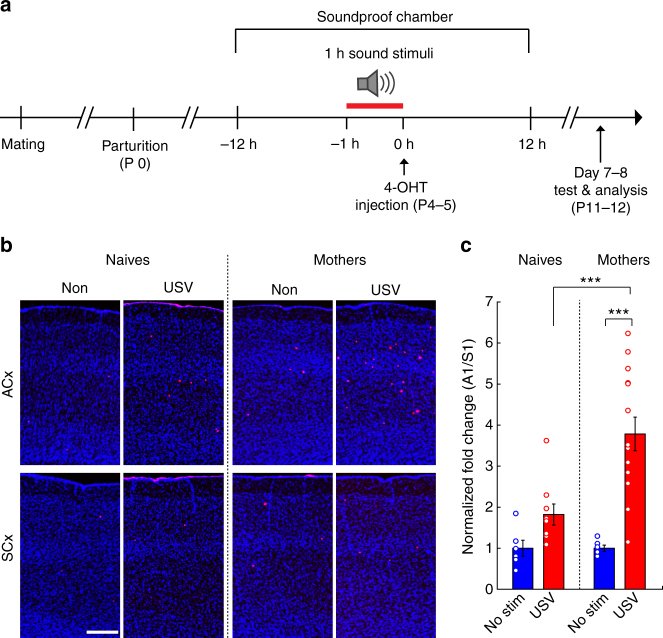
Recruitment of additional USV-responsive neurons in A1 of mothers. **a** The experimental protocol for TRAPing in mothers. **b** Representative fluorescent micrographs from the primary auditory (ACx, top rows) and somatosensory (SCx, bottom rows) cortices in naives and mothers. Each column shows images of ACx and SCx from the same mouse. The auditory stimulus is denoted above the images. **c** Quantification of the fold induction of TRAPed cells in A1 as compared to S1 (relative density), normalized to the ‘No Stim’ condition (mean ± SEM; Naives: same data as plotted in Fig. 1d; Mothers: ‘No Stim’ *N* = 6 mice, ‘USV’ *N* = 14 mice). USVs induced significantly higher number of the TRAPed cells than the relevant control group in naïves and mothers (***p < 0.001, post hoc Fisher’s LSD test after significant two-way ANOVA)

Next, we used TRAPxTB to study potential changes in encoding natural sounds in A1 during motherhood^13,31,32^. Given the different physiological/hormonal state of mothers, efficiencies of TRAP that are based on tamoxifen are expected to change. We thus re-calibrated TRAP in mothers using both TRAP and *Fos* staining. Since mothers had lower levels of baseline and induced Fos (Supplementary Fig. 7a–c; compare to Supplementary Fig. 3f), we used 25 mg/kg 4-OHT for our subsequent experiments. We TRAPed neurons in mothers with two sets of natural sounds 4–5 days after pups were born (P4–5), which is a period when mothers are highly engaged in parenting and when pups frequently emit calls. We played 1 h of sound stimulation in the mother’s home cage (when she was with her pups) and injected her with 4-OHT after sound stimulation (Fig. 4a). The TRAPing protocol had no effect on maternal behavior as assayed by pup retrieval assays before and after TRAPing (Supplementary Fig. 8). Mothers stayed in their cage and cared for their pups for another week after TRAPing and were then analyzed at P11–12 (Fig. 4a).

We tested whether WCs or USVs induced any changes in the number of neurons TRAPed in A1 of mothers, comparing three new experimental groups: (1) WC-stimulated mothers, (2) USV-stimulated mothers and (3) mothers that we did not play any external sounds to and were used as controls (*N* = 6, 14 and 6 mice, respectively). These three groups were also compared to the three matched experimental groups of naive mice (shown in Fig. 1c; Supplementary Fig. 9). Remarkably, USVs were now the most efficient stimulus in mothers as evident by >3.6-fold change in the number of TRAPed neurons in A1 (Fig. 4b, c). As compared to naive mice, this increase was particularly striking (Fig. 4c). WC stimulation also induced strong TRAPing in mothers but these were similar to those observed in naive females (Supplementary Fig. 9a, b). Together, these anatomical data suggest that the number of neurons representing USVs (but not WCs) is specifically increased in the auditory cortex of mothers.

### Transient highly USV-responsive neurons in mothers with TRAP

**Fig. 5:**
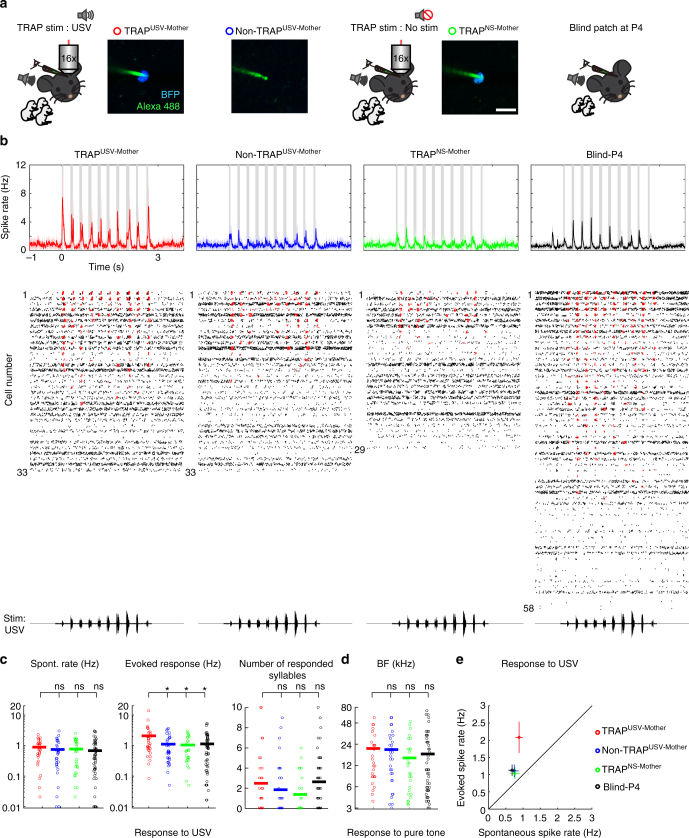
USV-TRAPed neurons in mothers are highly responsive to USVs. **a** Schematic representation of the four neuronal groups from three experimental conditions. We recorded from TRAPed and non-TRAPed neurons in P11–12 mothers stimulated with USV (TRAP^USV-Mother^ and non-TRAP^USV-Mother^, respectively), from TRAPed neurons in P11–12 mothers that were not stimulated with sound (TRAP^NS-Mother^, green) and from neurons in P4 mothers using blind patch configuration (Blind-P4, black). Example photomicrograph of the electrode (green) and BFP signal (blue) is shown from each group. Scale bar, 10 μm. **b** Spiking responses to the USV stimulus. Panels are organized similar to Fig. 3b, showing data from the following groups: TRAP^USV-Mother^ (*n* = 33 neurons, *N* = 10 mice), non-TRAP^USV-Mother^ (*n *= 33 neurons, *N* = 10 mice), TRAP^NS-Mother^ (*n* = 29 neurons, *N* = 7 mice) and Blind-P4 (*n* = 58 neurons, *N* = 7 mice). **c** Basic response properties to USV of all individual neurons from the four groups. Each circle represents an individual cell. USV TRAPed neurons have higher USV-evoked spike rates as compared to all control groups (*p < 0.05; post hoc Fisher’s LSD test after significant Kruskal–Wallis test). Spontaneous firing rates (*p* = 0.18, Kruskal–Wallis test) and the number of responded syllables (*p *= 0.12, Kruskal–Wallis test) were not significantly different between groups. **d** BF was not significantly different (*p* = 0.23, Kruskal–Wallis test) between the groups. **e** Plots of the mean (±SEM) evoked vs spontaneous spike rates of the neurons shown in (**b**) and (**c**)

Using loose patch recordings, we evaluated the spiking properties of the maternally recruited USV-TRAPed neurons. We compared four experimental groups of cells; the first three groups were from mothers TRAPed at P4–5 and recorded at P11–12: (1) TRAPed neurons in USV-stimulated mothers (TRAP^USV-mother^; *n* = 33 neurons, *N* = 10 mice), (2) non-TRAPed neighboring neurons in the same stimulated mothers (non-TRAP^USV-mother^; *n* = 33, *N* = 10) and (3) TRAPed neurons in non-stimulated mothers (TRAP^NS-mother^ *n* = 29, *N* = 7) (Fig. 5a). As expected, the neurons TRAPed by USVs in mothers responded significantly stronger to the USVs, as compared to the two control groups (Fig. 5b–d; compare red to the blue or the green peri-stimulus time histograms (PSTHs); Supplementary Tables 6 and 7). Notably, when the exact same comparison was performed in the naive group of mice, USV-TRAPed neurons showed only slightly higher (but not significantly different) responses as compared to the two controls (Supplementary Fig. 10).

Since TRAPing is carried out at P4 but recordings at P11, it remained possible that TRAPing is not specific to the calls we played at P4 but simply represents some highly active state of the whole population during P4, which then recedes at P11. To test this possibility, we determined population response profiles from a fourth group of cells, blindly patched neurons from P4 mothers (Fig. 5a; Blind-P4; *n* = 58, *N* = 7). The distribution of responses at P4 was similar to those of non-TRAPed neurons at P11, ruling out the above-mentioned possibility (Fig. 5b–e). Further, TRAPing experiments in mothers using WCs induced recombination in neurons that were as responsive as WC-TRAPed neurons in naive mice (Supplementary Fig. 9c, d, Supplementary Table 2). This further strengthens the argument that the stimulus itself (i.e., USVs) rather than the state of the animal is an effective inducer in TRAP. Taken together, using TRAP we reveal the recruitment of a subpopulation of USV-responsive neurons during motherhood.

**Fig. 6:**
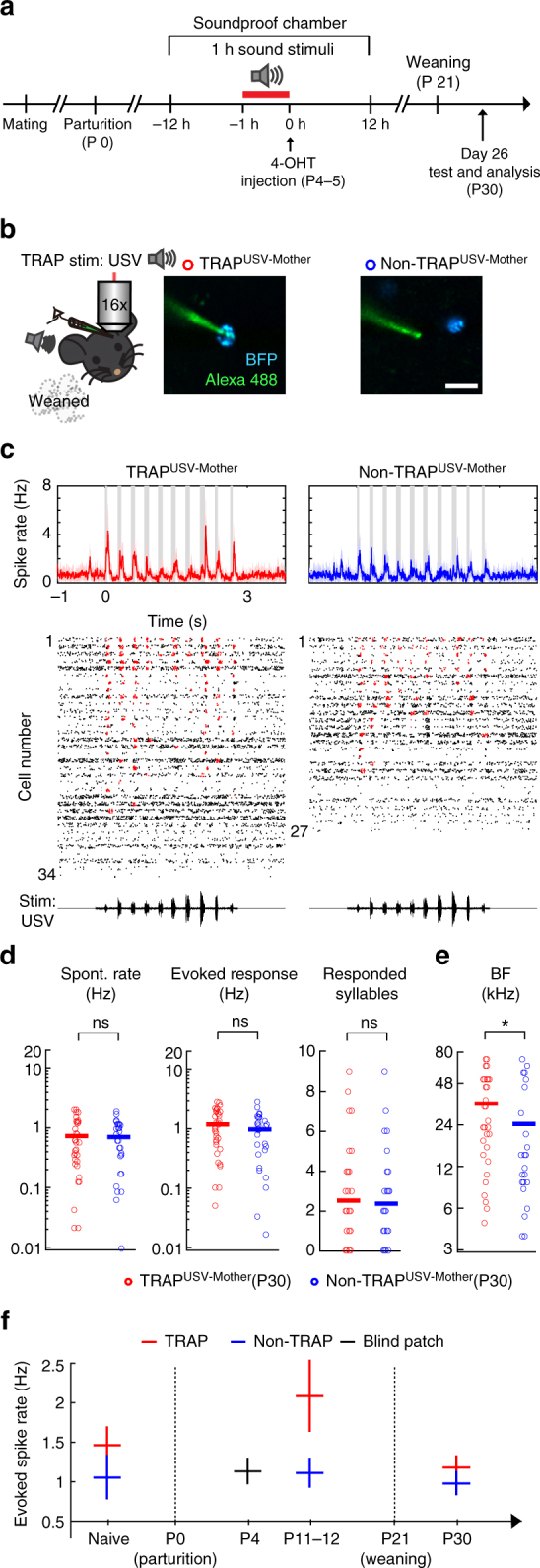
USV-TRAPed neurons of mothers are highly active only during motherhood. **a** The experimental protocol. **b**–**e** Plots organized the same as in Fig. 5a–d. **c** Spiking responses from the two neuronal groups from mothers TRAPed at P4 and recorded at P30, after pups were weaned (TRAP^USV-Mother^; *n* = 34 neurons, *N* = 6 mice, non-TRAP^USV-Mother^;* n* = 27 neurons, *N* = 6 mice). **d** We found no significant differences in spontaneous firing rates, USV-evoked spike rates and the number of responded syllables between groups (*p* = 0.96, *p* = 0.32, *p* = 0.76, respectively; Mann–Whitney *U*-test). **e** TRAP^USV-Mother^ neurons from our dataset had higher average BFs as compared to non-TRAP^USV-Mother^ neurons (*p < 0.05, Mann–Whitney *U*-test). **f** Collated summary of the evoked spike rates in response to USV at the different time points before and after motherhood (mean ± SEM; integrated from Supplementary Fig.10, Fig. 5, and this figure)

Taking advantage of the permanent labeling of the TRAP system, we next followed the temporal dynamics of USV-recruited neurons, asking whether USV-recruited neurons at P4 still remain highly active after the weaning of the pups. We TRAPed mothers with USVs at P4 and recorded from A1 at P30, 9 days after weaning of the pups (Fig. 6a). We recorded from two neuronal groups in P30—TRAPed neurons and their non-TRAPed neighbors (Fig. 6b; TRAP^USV-mother^; *n* = 34, *N* = 6; non-TRAP^USV-mother^; *n* = 27, *N* = 6). Interestingly, USV-TRAPed neurons so highly responsive in nursing dams were no longer overly responsive after weaning (Fig. 6c–e). This result suggests that maternal plasticity involves a transient recruitment of a subpopulation of highly USV-responsive neurons in A1 (summarized in Fig. 6f).

We next asked whether morphological differences could account for the transient plasticity in the TRAPed neurons by utilizing the advantage of the TB cassette discussed above (Supplementary Fig. 5c). We injected *AAV-TRE3G-GFP* to A1 of mice before the experiment and compared stimulated versus non-stimulated TRAPed neurons in mothers (Supplementary Fig. 11a). TRAPed neurons now expressed GFP strong enough to detect spines and axonal boutons with high resolution (Supplementary Fig. 11b, c). Quantitative morphological analysis showed that the transient recruitment of USV-responsive L2/3 neurons in mothers is not accompanied by morphological correlates as assessed by dendritic spine density or size, and axonal bouton density in their L5 axonal branches (Supplementary Fig. 11d–g). Thus, constitutive morphological changes seem to be largely orthogonal to the heightened responsiveness of the USV-recruited population in mothers. Other mechanisms, such as short-term morphological dynamics or changes in intrinsic properties of these neurons, may play a more prominent role^33^.

### USV-TRAPed neurons improve the coding of pup USVs

USV stimuli in mothers (Supplementary Fig. 12), but not in mothers following weaning (Supplementary Fig. 13) or naive females (Supplementary Fig. 14 and Table 1), tended to recruit a subpopulation of neurons having faster and higher evoked responses to pure tones (Supplementary Fig. 12 and Table 4). Thus, the USV-responding neurons in mothers not only outnumbered USV-responding neurons in naive females, but were also characterized by shorter latency and more robust responses to pure tones. Given that inferring responses of natural sounds from pure tones response is not trivial^2,3^, we next tested response profiles of TRAPed neurons to an array of other natural sounds.

**Fig. 7:**
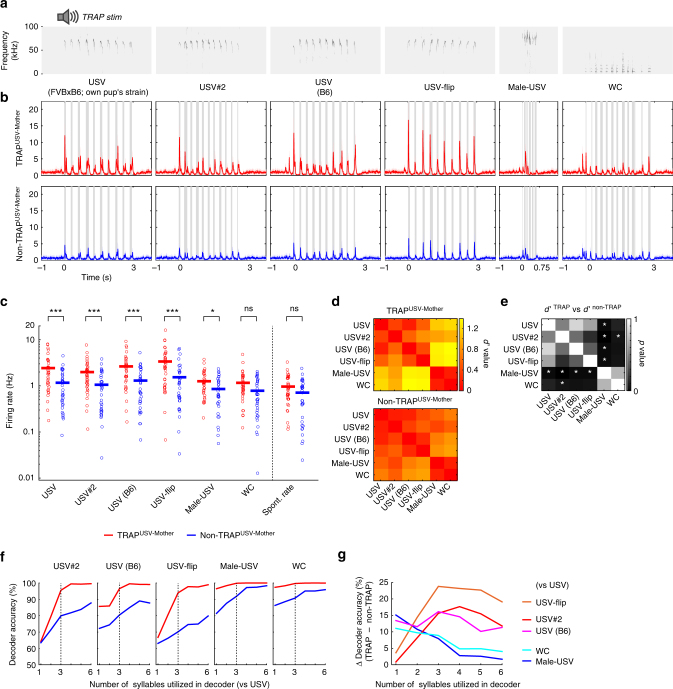
USV-TRAPed neurons in mothers are promiscuous to and improve coding of pup USVs. **a** Spectrograms of the 6 different sound stimuli used to test responsiveness in this experiment (see Methods for details). **b** Average PSTHs calculated from all neurons in response to the 6 sound stimuli from the two neuronal groups in this experiment (TRAP^USV-Mother^; *n* = 38 neurons (red), non-TRAP^USV-Mother^;* n* = 35 neurons (blue), *N* = 6 mice). **c** Spontaneous and evoked response properties of all neurons from the two groups (each circle represents an individual cell and the line indicates the mean). Neurons TRAPed with USVs (TRAP^USV-Mother^, red) respond higher to a collection of USV’s and particularly the different variations of pup-USVs (*p < 0.05; ***p < 0.001; ns, not significant, post hoc Fisher’s LSD test after significant Kruskal–Wallis test). Spontaneous firing rates were not significantly different (*p* = 0.09, Mann–Whitney *U*-test). **d** Matrices describing the average discriminability power of individual neurons between all combinations of different stimuli. Each pixel indicates the average *d*’ value calculated from the evoked spike rate from all neurons to two different calls. Both groups have similar trend but TRAPed neurons have better discrimination power between pup USVs and male-USV or WC (bottom left of matrix). **e** *P*-values of a Mann–Whitney *U*-test comparing between the values of each pixel of the matrices shown in (**e**). Asterisks indicate *p*-values lower than 0.05. **f** Classification performance of a Support Vector Machine (SVM) decoder. The decoder was tested for its accuracy to differentiate between any one of 5 different stimuli against the TRAP stimulus (USV (FVBxB6)). The performance of the decoder is shown for the two different groups separately (red, TRAP^USV-Mother^; blue, non-TRAP^USV-Mother^) and plotted against the number of syllables it was trained on and allowed to use. After 3 syllables (dotted lines) TRAPed neurons reached near 100% accuracy in decoding all pairs. **g** A plot of the difference in decoding performance between the TRAP^USV-Mother^ and the non-TRAP^USV-Mother^ (red–blue in (**f**)). The difference between the groups is more evident for the pup-USVs

In a separate set of experiments, we TRAPed mothers using the protocol described above (Fig. 4a) by playing USVs recorded from pups of the *TRAPxTB* strain (FVB/B6; F2 background). We then recorded the spiking responses from neurons at P11–12 in response to six different sounds (Fig. 7a). For each sound stimulus, we compared responses from two populations of neurons—TRAPed and non-TRAPed neighbors (TRAP^USV-mother^; *n* = 38, non-TRAP^USV-mother^; *n* = 35, *N *= 6 mice). As expected, the TRAPing stimulus evoked markedly stronger responses in the TRAPed compared to the non-TRAPed neurons (Fig. 7b; left panel, compare red to blue PSTH). Interestingly, TRAPed neurons showed low selectivity to other pup USVs. TRAPed neurons strongly responded to pup USVs regardless of the strain or the fine temporal sequence of the stimulus. There was, nevertheless, gross selectivity in the response profile. Specifically, TRAP neurons responded stronger to pup USVs as compared to the other natural calls, a property that was not shared by non-TRAPed neurons (Fig. 7b–c). To examine specificity more quantitatively, we calculated the discriminability of individual neurons to the different stimuli using *d*′, which describes the differences in response profiles of a neuron between any two calls. A low *d*′ value denotes high similarity (*d*′ = 0 is the exact same response intensity to two different calls), and high *d*′ denotes differential responsiveness. Neurons responded similarly to all pup calls and differentially to WC and male-USV (Fig. 7d), a property that was stronger in the TRAPed neurons (Fig. 7e).

Finally, we evaluated the information contained in the population rather than in single neurons by calculating classification accuracy of a decoder (see Methods). We used Support Vector Machine to test how the population classifies between the TRAPing stimulus (USV) versus all other stimuli. TRAPed neurons always performed better than non-TRAPed neurons, reaching near perfect classification after training from the first 3 syllables (Fig. 7f, dashed vertical lines). While all cells easily discriminated USVs from WC or male USV, the largest differences between TRAP and non-TRAP neurons were evident in classification among the more similar pup-USVs (Fig. 7g). These results support the argument that TRAPing tagged a subpopulation of cortical neurons with higher potential to contribute to the coding of pup USVs.

## Discussion

The anatomical organization of neural circuits in the cerebral cortex is often complex, with different functional units spatially intermingled^34^. To date, it remains technically difficult to target neuronal sub-networks that are predominantly active to specific stimuli for further experimentation. This challenge is particularly grand in the auditory cortex, which harbors complex functional architectures and sparse population codes^15^.

An increasingly popular strategy for genetically targeting active populations is based on conditional expression of transgenes driven by IEG promoters^26^. IEG-based methods can be noisy given that neurons are spontaneously active and have a large dynamic range of firing rates and temporal response profiles. Moreover, the slow timescales of gene expression (tens of minutes to hours) are not easily matched with the fast timescales by which neurons encode information (sub-seconds). *Fos* expression and firing patterns of a neuron are both functions of time-varying input to the cell, but these functions are not the same, which is why firing pattern and Fos activation cannot be directly inferred from each other. Given the wide usage of *Fos* activation as proxy for physiological events, it becomes critical to determine this relationship in specific brain regions and experimental designs but this has been attempted only in few cases^35–38^. As we show here, it is possible to determine the extent of this important correlation, which will set boundary conditions for any experiment that uses similar methods.

In A1, the sounds that were efficient in inducing TRAPing were not strictly correlated with higher evoked responses. For example, pure tones induce stronger evoked responses in A1 than natural sounds (Supplementary Tables 1 and 2), yet natural sounds were more efficiently TRAPed as compared to pure tones. We speculate that firing rates are not the only property that correlates with *Fos* expression. Rather, other response properties such as subthreshold input, the duration of a response and temporal dynamics could also contribute to the more prominent *Fos*-based expression in specific neurons. In A1, this would account for natural sounds inducing a pattern of activity that is more integrative in nature. Specifically, natural sounds drive combinations of frequencies rather than narrower band responses such as induced by pure tones. This is consistent with the expected role of A1 to encode sound features rather than spectral content alone^4^.

Building upon the strict tonotopic map in the cochlear nucleus, our previous study showed that *Fos* TRAP faithfully represents stimulus specificity^25^. Cortical responses, however, are more sluggish and sparse as compared to the brainstem, and the functional architecture of the cortex is more complex^39^. Since A1 is a challenging bench test for TRAP, our direct electrophysiological measurements are an important starting point to evaluate TRAP in terms of stimulus specificity in other cortical areas. Still, there are at least four sources of noise that contribute to our evaluation of TRAP. First, TRAPing was done during times when mice were unperturbed in their home cage, while recordings were done on anesthetized mice. Any differences between the awake and anesthetized states will contribute to differences in our measurements. Second, TRAPing and validation were carried out 7–8 days apart. It is unclear whether the same stimulus would activate the exact same cohort of neurons at different times; any such differences will also contribute to noise in validation. Third, a CreER-induced recombination event in a given cell is probabilistic and so we are TRAPing a subset of the actual active neurons in the network. Fourth, the stimulations used during TRAPing are an hour-long continuous replay of pup calls. This stimulus is played outside the direct context of the maternal behavior and is unlikely to occur in a natural context. The extent to which natural sounds themselves drive maternal plasticity remains unknown.

The new reporter mouse (TB) has several unique features as compared to existing viral or transgenic reporters. An advantage of TRAPxTB compared to all-viral based systems or other mice^22–24,36,38,40–43^ is that active neurons in the entire brain are TRAPed simultaneously. As a single copy transgene, it may also report the efficiency of recombination more consistently compared to AAV-based reporters that have variable copies. Furthermore, TB provides a means to genetically manipulate active neurons by virtue of permanently expressing tTA2 after recombination. Viruses utilizing a TRE reporter for gene expression can be injected locally to further study the neurons after recombination (Supplementary Fig. 5c, 11). Finally, TB can be used in combination with any Cre driver for studying different brain regions or cell types in a Cre-dependent and tTA2-dependent manner.

Several recent studies have highlighted the auditory cortex in the maternal brain as a site of plasticity. Plastic changes in the maternal brain include (but are not limited to) increased reliability of neuronal responses to USVs^10,11,13,32,44^. Despite these changes, many other physiological parameters remain stable like the tonotopic representation of the cortical sheet and single unit responses to pure tones^12,45^. Our blind recordings and recordings from non-TRAPed cells across groups and conditions (which presumably represent a random sample of cortical neurons) provide further support to the general notion of cortical stability with respect to pure tones in mothers.

We demonstrate in mothers a substantial increase in the representations of the pup USV-responsive neurons but not WC-responsive neurons. WCs and USVs differ completely in their frequency range, which are not equally represented in the auditory system of mice. Low frequencies (from which WCs are composed) induce strong responses as early as the brainstem. Responses to higher frequencies (from which USVs are composed) are weak in the brainstem, but become overrepresented from the level of the inferior colliculus^46^. Our data do not rule out that changes of WC-related activity may be found elsewhere along the auditory hierarchy, as suggested by some histological studies^7^.

Focusing on USV responding neurons, we found that TRAPed neurons were promiscuous to other pup-USVs (Fig. 7). This promiscuousness may have been expected from the results of the pure tones, whereas TRAPed neurons were generally more responsive (Supplementary Fig. 12 and Table 4). However, the particularly strong increase to pup USV as compared to male USVs suggest an additional form of increased sensitivity, perhaps in the way these neurons respond in the temporal domain. In fact, USV-TRAPed neurons in A1 of mothers carried similar amount of information towards the decoding of pup-USVs that are different in various spectro-temporal aspects of the stimulus. Based on our decoder analysis, we suggest that plastic changes in the mother’s brain increase the ability of the cortex to detect pup vocalizations but the code is redundant with respect to other pup calls and their variations. Although mothers will retrieve and nurse alien pups, they are nevertheless able to discriminate between the different calls^47^. A1, therefore, may be playing a role in generally detecting pup calls without fine discrimination, which may then occur in higher brain regions.

Naives and mothers are different animals in many respects and are not easily compared^14^. Eventually, the behavioral and physiological manifestation of motherhood is geared towards caring of the pups, which continue to grow and develop with changing needs. The dynamic nature of this transition in mothers is multisensory but remains poorly understood, particularly with respect to sensory coding and cortical plasticity. A recent report, studying plastic changes in A1 due to learning, showed a transient expansion of salient sounds, arguing that plasticity is necessary for learning but not for performance^48^. In this context, mothers following weaning will retrieve pups but slower than a lactating mom^44^. Thus, our finding of the transient nature of USV-responsive neurons during this critical postpartum period correlates well with the behavior of the dam. This transient plasticity in A1 could still be utilized by downstream circuits for learning that induce more permanent change that improves performance, similar to other learning mechanisms.

What could be the mechanisms for the maternal plasticity we observe? Evidence suggests that neuromodulation plays an important role. For example, oxytocin release from the paraventricular hypothalamic nucleus increases the response reliability to USVs by coordinating excitatory/inhibitory balance in the auditory cortex of mothers^13^. The noradrenaline system has also been implicated in maternal behaviors^49^, and generally in A1 plasticity^50^. Further, maternal behaviors are multisensory in nature and involve numerous brain regions, both cortical and subcortical^14,31,44^. The effects of neuromodulation in other brain regions (e.g., oxytocin also impacts the olfactory system^51^) support neuromodulation as a central candidate in parental plasticity. TRAPxTB offers a tool that can guide mechanistic studies underlying coding and plasticity in various regions of the mammalian brain.

## Methods

### Animals

All experimental procedures were approved by the Hebrew University Animal Care and Use Committee. *Fos-CreER*^*T2*^ (ref. ^25^) and *Ai14*^29^ were obtained from the Jackson laboratories (Background strain C57BL/6). TB mice were generated as detailed below (background strain FVB). We used the following mouse strains: *Fos-CreER*^*T2*^*;TB* double heterozygous female mice (F1 hybrid of C57BL/6 and FVB strain, 8–15 weeks old), *TB* heterozygous female mice (F1 hybrid of C57BL/6 and FVB strain, 9–10 weeks old), wild-type female mice (F1 hybrid of C57BL/6 and FVB strain, 9–10 weeks old) and *Fos-CreER*^*T2*^*;Ai14* double heterozygous female mice (C57BL/6 strain, 8–10 weeks old).

### Generation of the TB mouse

A *Pac*I-*Not*I fragment containing a *CAG-loxP-Stop-loxP-TdTomato-WPRE-SV40pA* cassette from the Ai9 targeting construct (Addgene Plasmid #22799)^29^ was ligated into the backbone of *pBT378_pattB-pCA-GFP-pA-attB* (Addgene Plasmid #52554)^27^. The resulting intermediate plasmid was digested with *Bam*HI-*Mlu*I, and a synthetic fragment (Genscript) containing *tTA2-2A-H2B-TagBFP-3xmyc* and necessary linker sequences was inserted to obtain the final *pBT378-TB* construct. This construct was tested in vitro by transient transfection into 293T cells with a mammalian Cre expression construct and a *TRE-GFP* reporter construct (Supplementary Fig. 1). *pBT378-TB* was microinjected into the pronuclei of H11P3 zygotes^27^ by the Stanford Transgenic Facility. Founders were screened by PCR for cassette exchange of the *TB* cassette into the *H11* locus, and positive mice were bred to establish the colony.

### DNA constructs

*TRE-GFP*, *TRE3G-GFP*, *TRE-MT*^*66T*^*G* (a gift from Kazunari Miyamichi) and *TRE3G-ChETA-mCherry* were constructed using standard molecular cloning methods based on polymerase chain reaction (PCR) and restriction enzymes commercially available from New England Biolabs. To make the AAV containing the *TRE-GFP* cassette, TRE promoter from *pAAV-TRE-HTG* (Addgene Plasmid #27437)^52^ was subcloned into *pAAV-CMV-GFP*, digested with *Mlu*I and* Age*I. *pAAV-CMV-GFP* was obtained by subcloning of cytomegalovirus (CMV) promoter and GFP into *pAAV-hSyn-NpHR3.0-YFP* (Addgene Plasmid #26972; a gift from Karl Deisseroth) digested with *Mlu*I and *Eco*RI. To make the AAV containing the *TRE3G-GFP* cassette, *TRE3G* (Clontech), *GFP* and the *WPRE* were subcloned using an InFusion kit (Clontech) simultaneously in *pAAV-TRE-HTG* (Addgene Plasmid #27437)^52^, digested with *Mlu*I and *Bgl*II. To make AAV containing the *TRE-histone2B-mCherry-2A-TVA66T-2A-Rabies-Glycoprotein* (TRE-*MT*^*66T*^*G*) cassette, mCherry and TVA^66T^ (ref. ^30^) were subcloned into *pAAV-TRE-HTG* (Addgene Plasmid #27437)^52^. To make the AAV containing the *TRE3G-ChETA-mCherry* cassette, ChETA-mCherry amplified by PCR from *pAAV-CaMKIIa-hChR2(E123T/T159C)-mCherry* (Addgene Plasmid #35512)^53^ was subcloned into *TRE3G-GFP* digested with *Nco*I and* Eco*RI.

### Viral procedure

AAV vectors containing *TRE-GFP* (1 × 10^12^ genomic copies per ml), *TRE3G-GFP* (2 × 10^13^ genomic copies per ml), *TRE-MT*^*66T*^*G* (1 × 10^12^ genomic copies per ml) and *TRE3G-ChETA-mCherry* (4 × 10^12^ genomic copies per ml) were produced by the Hebrew University viral vector core. Then, 0.2 μl of *AAV2-TRE-GFP or AAV2-TRE3G-GFP* was stereotaxically injected into the left auditory cortex (coordinates relative to Bregma: anterior 2.5 mm, lateral 4.5 mm, depth 1.85 mm with 25 degree) by using Nanoject (Drummond Scientific). EnvA-Pseudotyped RabiesΔG (3 × 10^11^ infectious particles per ml) was produced following the established protocol^54,55^. For trans-synaptic tracing from TRAP cells, 0.1 μl of *AAV2-MT*^*66T*^*G* was injected to the left auditory cortex (same coordinates as above). Next, 0.5 μl of *AAV9-TRE3G-ChETA-mCherry* was injected to the left auditory cortex at two different depths (coordinates relative to Bregma: anterior 2.5 mm, lateral 4.5 mm, depth 1.6 and 2.0 mm with 28 degree).

### Drug preparation

The 4-hydroxytamoxifen (Sigma-Aldrich, Cat#H6278) was dissolved to 20 mg/ml in ethanol by shaking at 37 °C for 15 min, then aliquoted and stored at −20 °C for up to several weeks. Before use, 4-OHT was re-dissolved in ethanol by shaking at 37 °C for 15 min. Corn oil (Sigma-Aldrich) was added to give a final concentration of 2 or 4 mg/ml, and the ethanol was evaporated by vacuum under centrifugation. The final 2 or 4 mg/ml 4-OHT solutions were stored at 4 °C before use (for no more than 24 h). All injections were made intraperitoneally. A total of 25 or 30–35 mg/kg 4-OHT were delivered for the experimental groups of TRAP^NS^ or TRAP^NS-mother^, respectively. We worked with 15 mg/kg 4-OHT in naive mice after a thorough calibration to achieve near zero noise levels. This dose was also chosen because our study targeted a sparse application. Note, however, that higher doses could reach up to 38% of the potentially maximal induction as assessed by Fos staining (Supplementary Fig. 3f; 50 mg/kg). Higher doses means higher noise but similar TRAPing efficiency (Supplementary Fig. 3c). Thus, in other applications where higher number of neurons are desirable, higher doses may be adjusted. We also worked with a slightly higher dose of 4-OHT in mothers, because the induction appeared to fail in most of mothers administrated with 15 mg/kg 4-OHT.

We waited for 7–8 days between TRAP and analysis because the full expression of the transgenes from *Fos*TRAP requires several days after the injection of 4-OHT and we gave mothers 7–8 days of maternal experience while the pups are still completely dependent and maternal care is high.

### Auditory stimulation for TRAP

Sound stimulation and injection of 4-OHT were conducted in a soundproof chamber (IAC Acoustics). Sound stimuli were custom-generated in MATLAB (MathWorks) and delivered by a free field speaker (EC1, Tucker-Davis Technologies) placed above the home cage. For pure tone stimulation, 6 kHz or 24 kHz pure tones were played for 1 h (total of 3600 repetition of 100 ms duration at 78 dB sound pressure level (SPL), 10 ms linear onset and offset ramps and interstimulus-interval (ISI; offset to onset) of 900 ms). For natural sound stimulation, WCs or USVs were delivered for 1 h (total of 900 repetition of 3 s duration and ISI of 1 s). WCs (from C57BL/6 strain) and USVs (from C57BL/6 and F2 hybrid of C57BL/6 and FVB strain; used in Fig. 7) were recorded with a one-quarter inch microphone (Brüel & Kjær) from P4–P5 pups. Male-USV was recorded from an adult male mouse (F1 hybrid of C57BL/6 and FVB strain) in the presence of female naive mouse. Vocalizations were sampled at 500 kHz and identified offline (Digidata 1322 A; Molecular Devices). USVs were identified offline and cleaned from noise using OM-LSA algorithm^56^. To make USV-flip sound protocol, the time window for each syllables in USV (FVBxB6) were identified and the sound signals within each time window were reversed. Two different pup’s USVs from F2 hybrid of C57BL/6 and FVB strain were used in Fig. 7 (denoted as USV(FVBxB6) and USV#2). WC, USV (B6), USV (FVBxB6), USV#2 (FVBxB6) and male-USV contained 13, 10, 11 and 6 syllables, respectively. Attenuations of WCs and USVs were adjusted to match at the lowest attenuation level (USVs was attenuated by ~10 dB SPL more than WCs).

### Two-photon targeted recordings from TRAPed neurons

Animals were anesthetized with an intraperitoneal injection of ketamine and medetomidine (80 mg/kg and 0.65 mg/kg, respectively) and a subcutaneous injection of Carprofen (0.004 mg/g). Additionally, dextrose-saline was injected to prevent dehydration. The depth of anesthesia was assessed by monitoring the pinch withdrawal reflex and ketamine/medetomidine was added to maintain it. The animal’s rectal temperature was continuously monitored and maintained at 36 °C ± 1 °C. For imaging and recording, a custom made metal pin was glued to the skull using dental cement, and connected to a custom stage to allow precise positioning of the head relative to the speaker (facing the right ear). The muscle overlying the left auditory cortex was removed and a craniotomy (~2 × 2 mm) was performed over A1 (which was verified anatomically and physiologically for each mouse) as previously described^20^.

Imaging was performed using an Ultima two-photon microscope from Prairie Technologies (Middleton, WI), equipped with a 16× water-immersion objective lens (0.8 NA;CF175, Nikon). Two-photon excitation of the electrode and somata simultaneously was used at 840 nm (DeepSee laser, Spectra-physics). A1 here refers to both A1 and anterior auditory field, which were not separated in our measurements.

Electrodes (4–8 Mohm) were pulled from filamented, thin-walled, borosilicate glass (outer diameter, 1.5 mm; inner diameter, 1.0 mm; Hilgenberg) on a vertical two-stage puller (PC-12, Narishige). Internal solution contained the following (in mM): 140 K-Gluconate, 10 KCl, 10 HEPES, 10 Na_2_-Phosphocreatine, 4 MgATP, 0.4 Na_2_GTP, 0.5 EGTA adjusted to pH 7.25 with KOH. For electrode visualization we supplemented the internal solution with 100 μM Alexafluor 488 (Invitrogen). To minimize pulsations we placed 1.5% low melting agar (type 3a, Sigma-Aldrich) over the craniotomy.

We acquired all recordings using an intracellular amplifier in current clamp mode (Multiclamp 700B, Molecular Devices) at a sampling rate of 10 kHz (Digidata 1440 A, Molecular Devices). We filtered all recordings using a 100 Hz high-pass filter.

### Auditory stimuli for electrophysiological recording

The natural call protocol was presented 20 times at 3 attenuation levels (WCs: 0, 10 and 20 dB attenuation; USVs: 0, 15 and 30 dB attenuation). In Fig. 7, the natural call protocol was presented 20 times at 3 attenuation levels (0, 15 and 30 dB attenuation) for all natural sound stimuli. The highest intensity (0 dB attenuation) was adjusted to be as the same sound intensity as the stimuli played during TRAPing. Interstimulus interval was 1 s. The frequency-response area (FRA) protocol was composed of 30 pure tones from 3–40 or 3–80 kHz (100 ms duration, 3 ms on and off linear ramps) logarithmically spaced and presented at four SPLs (78–48 dB SPLs). Each stimulus/intensity combination was presented 12 times at ISI of 600 ms (total of 1440 stimuli presented pseudo-randomly).

The following stimuli were used in the experiment in Fig. 7. ‘USV’ is a recorded call from a pup with FVBxB6 background (also used as the TRAPing stimulus in this experiment); ‘USV#2’ is a different USV from the same strain; ‘USV(B6)’ is a call recorded from a pup with c57/B6 background (used as the TRAPing stimuli in previous figures); ‘USV-flip’ is the same as ‘USV (FVBxB6)’ but where each syllable is played in reverse; ‘male-USV’ is a call recorded from a male in the presence of a female; ‘WC’ is a low-frequency communication sound emitted by pups.

### Histology

Mice were given an overdose of Pental and were perfused transcardially with phosphate-buffered saline (PBS) followed by 4% paraformaldehyde (PFA) in PBS. Brains were post-fixed for 12–24 h in 4% PFA in PBS and then cryoprotected for >24 h in 30% sucrose in PBS. Then, 40 μm coronal slices of the entire cortex and in some cases the entire brain were made using a freezing microtome (Leica SM 2000R) and preserved in PBS. Free floating slices were then incubated in the following solutions with gentle agitation at room temperature: 2 h in blocking solution (5% heat inactivated goat serum, 0.4% Triton-X100 in PBS); overnight at room temperature or 3–4 nights at 4 °C (for staining with anti-Fos) in primary antibody 1:1000 mouse anti-Myc or rabbit anti-Myc (Santa Cruz, Cat #sc-40 or Abcam, Cat #ab9106, respectively) in blocking solution; 2–3 h in secondary antibody 1:500 goat anti-mouse-IgG Cy3-conjugated or goat anti-rabbit-IgG Cy3-conjugated (Jackson ImmunoResearch) in blocking solution; 15 min in 2.5 μg/ml of DAPI (4',6-diamidino-2-phenylindole) (Santa Cruz) in PBS. Sections were mounted on slides and cover-slipped with mounting media (Vectashield H-1000). The primary antibodies used in this study were mouse anti-Myc (1:1000 for overnight at room temperature or 1:2000 for 3–4 nights at 4 °C; Santa Cruz, Cat #sc-40), rabbit anti-Myc (1:1000; Abcam, Cat #ab9106) and rabbit anti-Fos (1:10,000; Synaptic Systems, Cat #226003). Secondary antibodies conjugated with Alexa 488 or Cy3 (Jackson ImmunoResearch) were diluted 1:500 from 50% glycerol stocks. Sections were imaged using an Olympus IX-81 epifluorescent microscope with a 4× and 10× objective lens (0.16 and 0.3 NA; Olympus) and Olympus FV3000 confocal microscope with 10× or 60× objective lens (0.4 and 1.4 NA, respectively; Olympus). Images were processed in Photoshop and ImageJ to adjust contrast and brightness of each channel.

For counting the number of TRAPed cells in A1 and S1, every 40 μm 12 coronal sections through most of the rostral-caudal extent of A1 and the forelimb region of S1 were imaged (Supplementary Fig. 4). We found no inter-hemispheric differences between left and right A1, and thus only the right A1 and one S1 (either right or left, chosen randomly) for each mouse were selected for quantitative analysis. The number of TRAPed cells and the volume of A1 and S1 in the counted sections were summed across all sections for each animal, and the sums were used to calculate the density of TRAPed cells in A1 and S1. The density of TRAPed cells in A1 was normalized to the density of TRAPed cells in S1 for each mouse. The experimental group of ‘No Stim’ was set to 1.

For counting the number of Fos positive cells in A1 and S1, we counted 12 coronal section, taken every 40 μm from the same regions as for counting the number of TRAPed cells. Slices were imaged using Olympus FV3000 confocal microscope at 5 μm steps in the Z dimension with 10× objective lens (0.4 NA; Olympus). The number of stained cells with Fos in the cortex form a continuous spectrum from weak to strong, and quantifying it becomes somewhat arbitrary as to where to draw the threshold. Thus, we set the certain threshold for all groups and the number of cells was automatically counted using custom-written code in MATLAB. Briefly, to correct uneven illumination and filter out the noise the raw images were processed by the MATLAB functions imtophat and medfilt2. Then, a binary mask was made from the pixels which had >50 brightness intensity (range; 0–255). The number of the cells was measured from this binary mask. Overlapped cells were separated by watershed function in MATLAB.

For counting the number of TRAPed cells in the auditory pathway throughout the brain (Supplementary Fig. 5a), all 60 μm coronal slices from the entire brain were immunostained as described above. Every 60 μm, 8 coronal sections through most of the rostral-caudal extent of A1, the forelimb region of S1, the thalamic region of medial geniculate body (MGB), 6 coronal sections through the brain stem region of IC and 12 coronal sections through the brain stem region of VCN were imaged. The left and right A1 and one S1, MGB, IC and VCN (either right or left, chosen randomly) for each mouse were selected for counting. The density of TRAPed cells in A1, MGB, IC and VCN were normalized to the density of TRAPed cells in S1 for each mouse. The experimental group of ‘No Stim’ was set to 1.

For counting the number of dendritic spines and axonal boutons in A1, 60 μm coronal sections were stained with anti-Myc and imaged using Olympus FV3000 confocal microscope at 0.5 μm steps in the Z dimension with 60× oil objective lens (1.4 NA; Olympus).

### Data analyses

Data analyses and statistics were performed using custom-written code in MATLAB. The raw voltage traces were high-pass filtered using the Axoclamp’s built-in filter at 100 Hz. Spikes were extracted from these filtered traces by thresholding. Spike times were then assigned to the local peaks of suprathreshold segments and rounded to the nearest millisecond. For each cell, we obtained a peri-stimulus time histogram (PSTH, binned at 1 ms) for both protocols (i.e., natural stimuli and pure tones).

For the natural stimuli protocol, evoked firing rates were extracted based on a constant time window of 100 ms following syllable onset. In Fig. 7, evoked firing rates were extracted based on time window of 100 ms or the same size as the length of the syllable, because several syllables have longer time window than 100 ms. The values presented for evoked firing rate of each cell is the mean value of the evoked firing rate to all the syllables. The spontaneous firing rate of the cell was calculated based on the average of all 800 ms preceding each natural sounds stimulus presentation (corresponding to −1 to −0.2 s in PSTH plots in figures). Significant response to each syllable was determined by the Mann–Whitney *U*-test of the firing rates based on the time window of the full-width-half-maximum (FWHM) of the PSTH compared to the cell’s spontaneous firing rate. Spikes within the time window of the FWHM of the PSTH that passed significance (*p* < 0.05) were marked as red in their raster plots for visual clarity. Only in Fig. 7, the cells which had no significant response to all 6 calls were excluded from our dataset (0 and 3 cells were excluded from the TRAPed and non-TRAPed groups, respectively).

For the pure tone protocol, the evoked response window was determined as the FWHM of the PSTH of the cell. We calculated six parameters for each cell as follows. (1) Spontaneous firing rate of the cell was calculated based on the average of all 100 ms preceding each stimulus presentation. (2) Evoked firing rate was calculated based on the evoked response window determined as above. (3) Response latency presented in the figures is the peak of PSTH. Of note, using other methods to calculate the latency like the time when the PSTH reached 2 SD above baseline did not change any of the conclusions. (4) BF is the tone frequency that elicited the strongest response across all intensities. (5) Characteristic frequency is the tone frequency that elicited the strongest response averaged across all intensities. (6) FRA matrices were composed of the mean evoked firing rates for each frequency and intensity. Each matrix was normalized by the maximum firing rate for that cell.

Quantitative analyses of the spine density were performed manually from the filtered image stacks (Gaussian blur filter using ImageJ). The number of dendritic spines on the apical dendrites of TRAPed cells in L2/3 were manually counted using imageJ Plugin ‘Cell Counter’. The length of the dendrites where the number of spines was counted was measured by ImageJ Plugin ‘Simple Neurite Tracer’. The distribution of spine head size was measured by custom-written program in MATLAB described previously^57^. Axonal boutons were counted manually from the axonal branches in Layer 5. The length of the axons was measured by ImageJ Plugin ‘Simple Neurite Tracer’.

### Calculation of *d*’

Discriminability power of individual neurons between different stimuli was computed using the metric *d*ʹ, which is the difference in mean of the two distributions normalized by the mean of the two standard deviations:

$$d{\prime} = \frac{{\mu _{{\mathrm{evoked}}\,({\mathrm{stim}}\# 1)} - \mu _{{\mathrm{evoked}}\,({\mathrm{stim}}\# 2)}}}{{\sqrt {\frac{1}{2}\left( {\sigma _{{\mathrm{evoked}}\,({\mathrm{stim}}\# 1)}}^2 + \sigma _{{\mathrm{evoked}}\,({\mathrm{stim}}\# 2)}^{2} \right)}}}$$

The stimulus-evoked spike count distribution was computed from the mean value of the evoked firing rate to all the syllables.

### Decoder analysis

Classification of call identity using population activity was done using the Support Vector Machine (SVM) classifier with a linear kernel. The input to a SVM consisted of the spike count of each neuron in the 100 ms or same length of the syllable (if the syllable has longer window than 100 ms) following syllable onset. Thus, a response vector to the stimulus is composed of each neuron’s spike count to each of the first syllables of a stimulus up to some number of syllables. In each run, 90% of the data (18 trials from the highest sound intensity) were used for training and 10% of the trials (2 trials from the highest sound intensity) were used as test for decoding accuracy. Each classifier was iterated 1000 times and a mean accuracy was calculated. Different combinations of trials were chosen randomly for each run. We increased the number of syllables cumulatively from 1 up to 6 for each sound stimulus to measure the accuracy at an increasing number of syllables. We ran 10 classifiers—2 populations (TRAP and non-TRAP), which compared a USV stimulus (FVBxB6; TRAPing Stim) against each of 5 different sound stimuli.

### Pup retrieval assay

Animals were placed in a new cage with standard wood chip bedding and a red transparent plastic mock nest in one corner and allowed 1 h of of free exploration. Five pups at postnatal day 3–6 were placed in the cage consecutively with 30–50 s intervals. The experiment was terminated when all pups were retrieved or after 5 min. The probability and the latency to retrieve pups were scored manually from the videotape.

### Data availability

The data that support the finding of this study are available from the corresponding authors upon reasonable request.

## Electronic supplementary material

Supplementary Information
