# Supplementary material for: Genetic tagging of active neurons in auditory cortex reveals maternal plasticity of coding ultrasonic vocalizations
Source: Nat Commun. 2018 Feb 28;9:871. doi: 10.1038/s41467-018-03183-2 (PMC5830453; doi:10.1038/s41467-018-03183-2)
Supplement: Supplementary file 1 — Supplementary Information [file 41467_2018_3183_MOESM1_ESM.pdf]

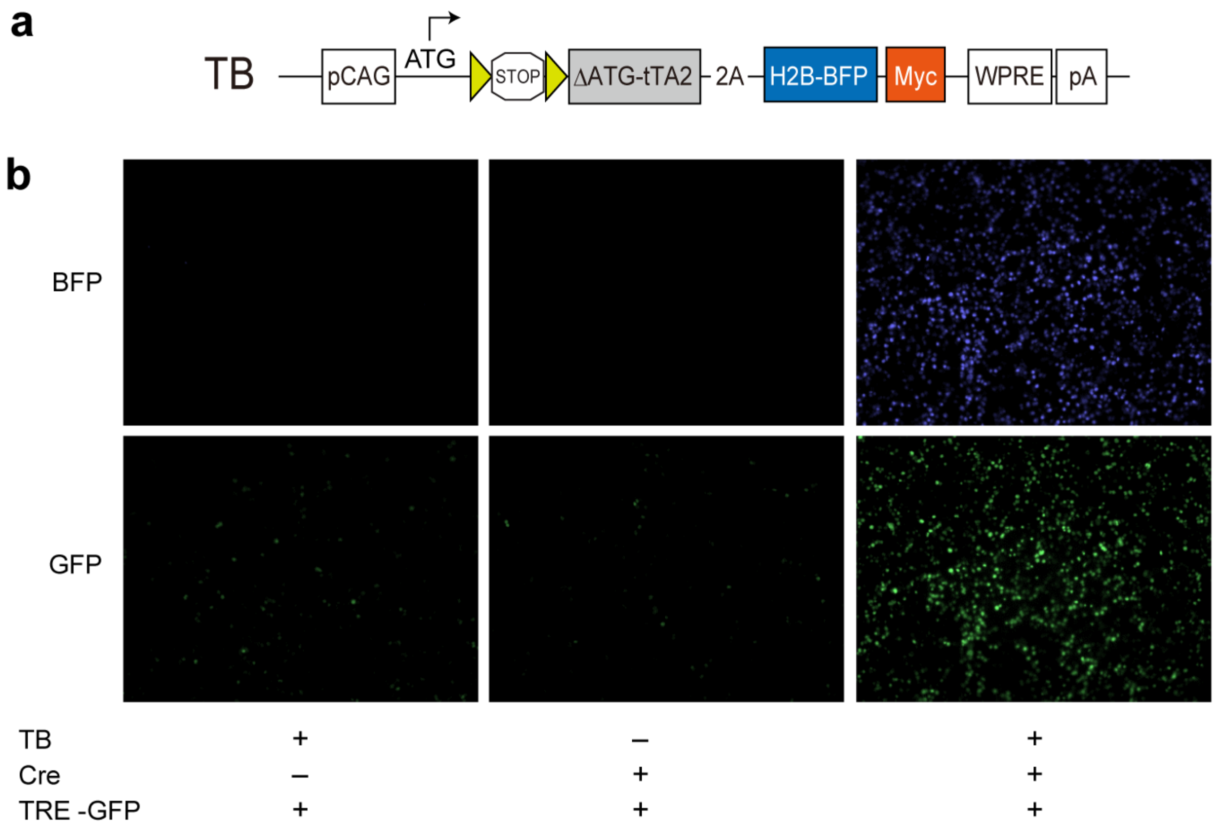

Supplementary Figure 1. Design of the TB transgene and *in vitro* validation

(a) The TB transgene consists of a *CAG* promoter/enhancer element, a *loxP-STOP-loxP* cassette, the tetracycline-dependent transactivator tTA2 linked by a 2A peptide to histone 2B (H2B) tagged with blue fluorescent BFP (BFP) and myc, a WPRE element to facilitate transgene expression[1], and a polyadenylation (pA) signal. The start codon was placed before the *loxP-STOP-loxP* cassette to reduce the amount of functional tTA2 produced by leaky transcriptional read-through of the STOP cassette. After Cre recombination, a small peptide from translation of the *loxP* sequence will be fused to the N-terminus of tTA2. The BFP was fused at its N-terminus to histone H2B to promote nuclear localization and at its C-terminus to a 3 x myc tag to facilitate immunostaining. **(b)** HEK293T cells were transfected with TB, a Cre-expression construct, and/or a construct in which GFP is driven by a tTA2-dependent regulatory element (TRE-GFP). In the absence of Cre (left), only a sparse population of cells weakly express GFP. Since similar GFP expression was observed when TB was omitted (middle), this expression is presumably due to leaky tTA2-independent transcription from the TRE rather than leaky expression of tTA2. In the presence of all three components, both GFP and BFP are highly expressed by many cells (right).

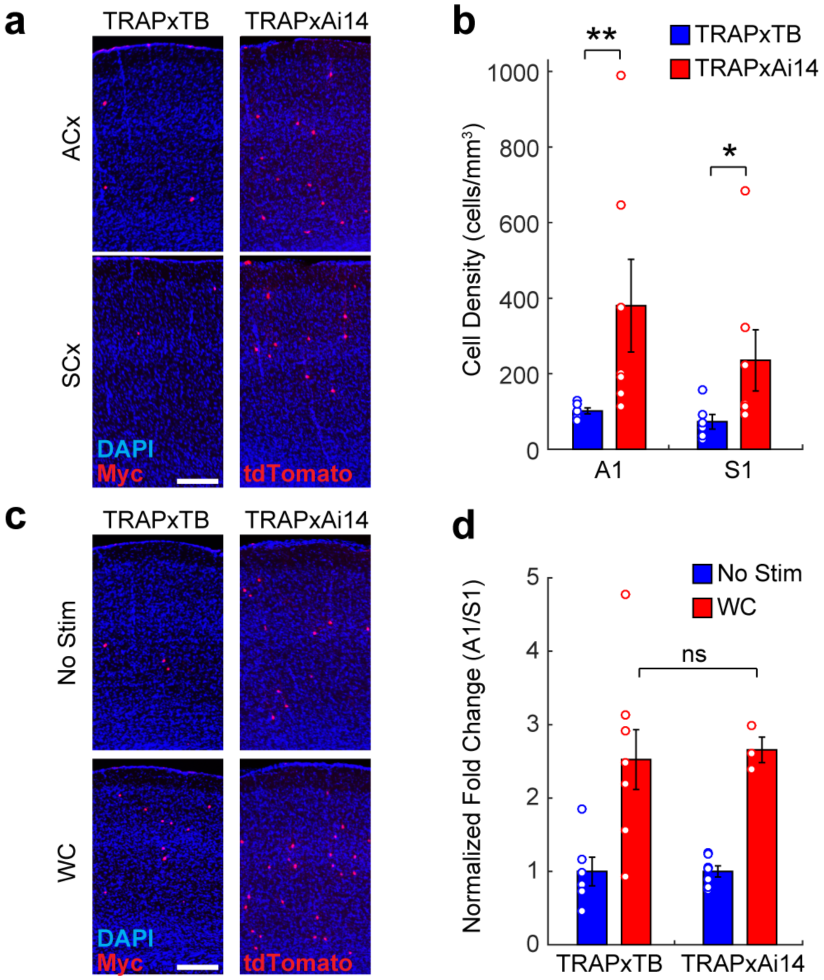

Supplementary Figure 2. TB has lower background noise but similar induction efficiency as Ai14

(a) Representative fluorescent micrographs of the primary auditory (ACx, top rows) and somatosensory (SCx, bottom rows) cortices in *TRAP x TB* (left) and *TRAP x Ai14* (right) without any sound stimulation. Each column shows images of ACx and SCx from the same mouse. Scale bar, 200 μm. **(b)** Quantification of the density of TRAPed cells in ACx and SCx (mean ± SEM; '*TRAP x TB*' N=6 mice, '*TRAP x Ai14*' N=7 mice). *TRAP x Ai14* had significantly higher number of the TRAPed cells than *TRAP x TB* (*, $p<0.05$; **, $p < 0.01$, Mann-Whitney U test). **(c)** Representative fluorescent micrographs of ACx of *TRAP x TB* (left column) and *TRAP x Ai14* (right column) without or with WC stimulation (rows). **(d)** Quantification of the density of TRAPed cells in A1 relative to their density in S1 and normalized to the 'No Stim' condition (mean ± SEM; '*TRAB x TB*': same data as plotted in Fig. 1d; '*TRAP x Ai14*': 'No Stim' N=7 mice, 'WC' N=3 mice). WC induced a significant increase in the number of the TRAPed neurons in A1 in both *TRAP x TB* and *TRAP x Ai14*. Induction efficiency was not significantly different between the groups (**, $p < 0.01$, ns, not significant, $p=0.80$, post hoc Fisher's LSD test after significant Two-way Anova test).

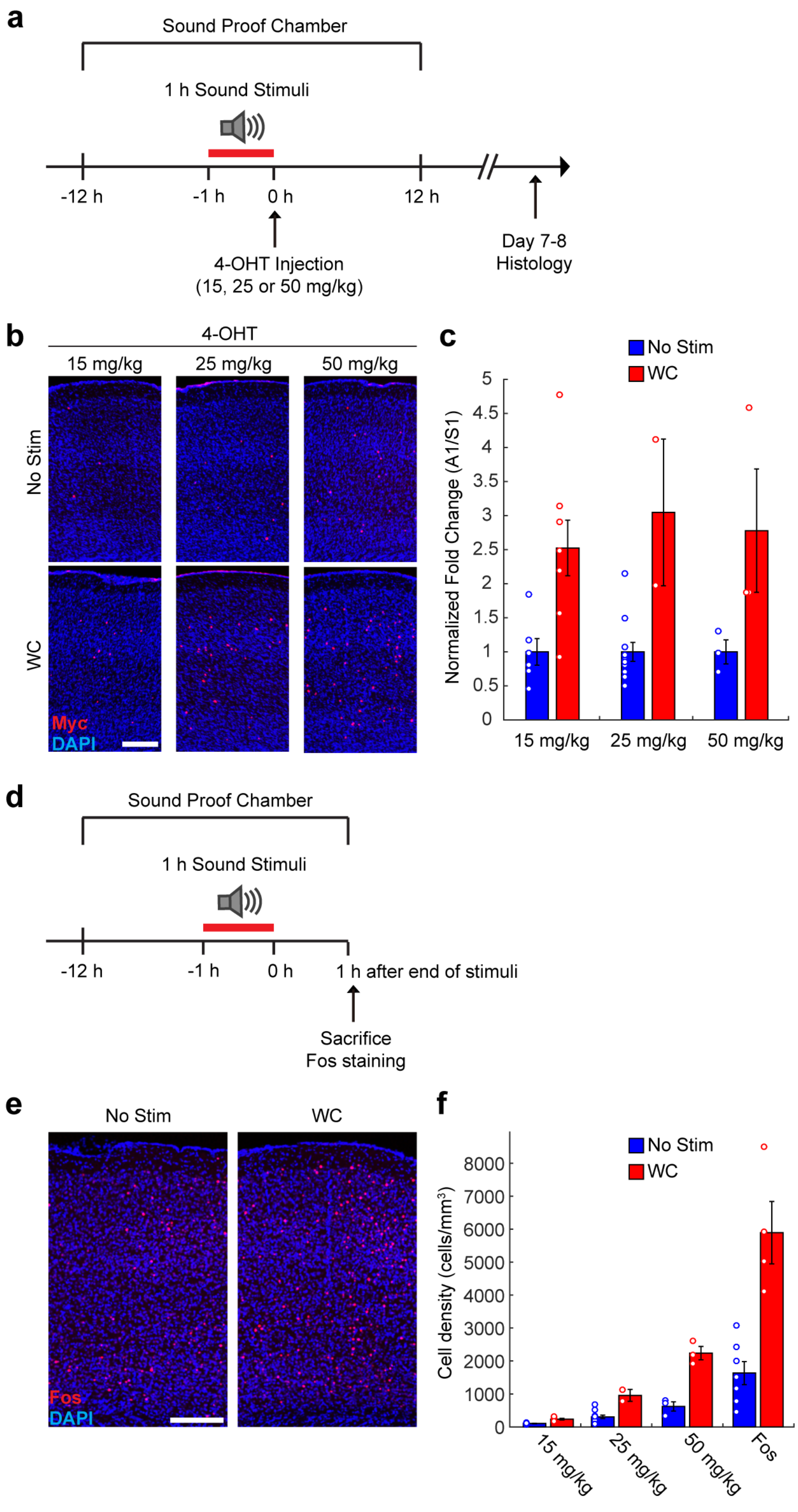

Supplementary Figure 3. Calibration of the TRAP x TB system: dose response curve and comparison to Fos

(a) The experimental protocol for calibrating TRAP with 3 different doses of 4-OHT injection. **(b)** Representative fluorescent micrographs of ACx in mice injected with the different doses of 4-OHT and without or with WC stimulation (columns: 4-OHT dose; rows: stimulus). Scale bar, 200 μm. **(c)** Quantification of the density of TRAPed cells in A1 relative to their density in S1 and normalized to the 'No Stim' condition (mean ± SEM; '15 mg/kg': same data as plotted in Fig. 1d; '25 mg/kg': 'No Stim' N=11 mice, 'WC' N=2 mice; '50 mg/kg': 'No Stim' N=3 mice, 'WC' N=3 mice). **(d)** The experimental protocol for Fos staining. **(e)** Representative fluorescent micrographs of the ACx stained with anti-Fos in mice stimulated with WC or not stimulated with sound. Scale bar, 200 μm. **(f)** Quantification of the density of TRAPed cells injected with 3 different doses of 4-OHT (same groups as shown in 'a' - 'c') and Fos positive cells in ACx (mean ± SEM; 'Fos': 'No Stim' N=7 mice, 'WC' N=4 mice). The densities of TRAPed cells were lower than the density of Fos positive cells. The proportions of the 3 doses (15, 25, and 50 mg/kg) relative to Fos were 6, 19, and 38% in 'No Stim' condition and 4, 16, and 38% in the 'WC' condition.

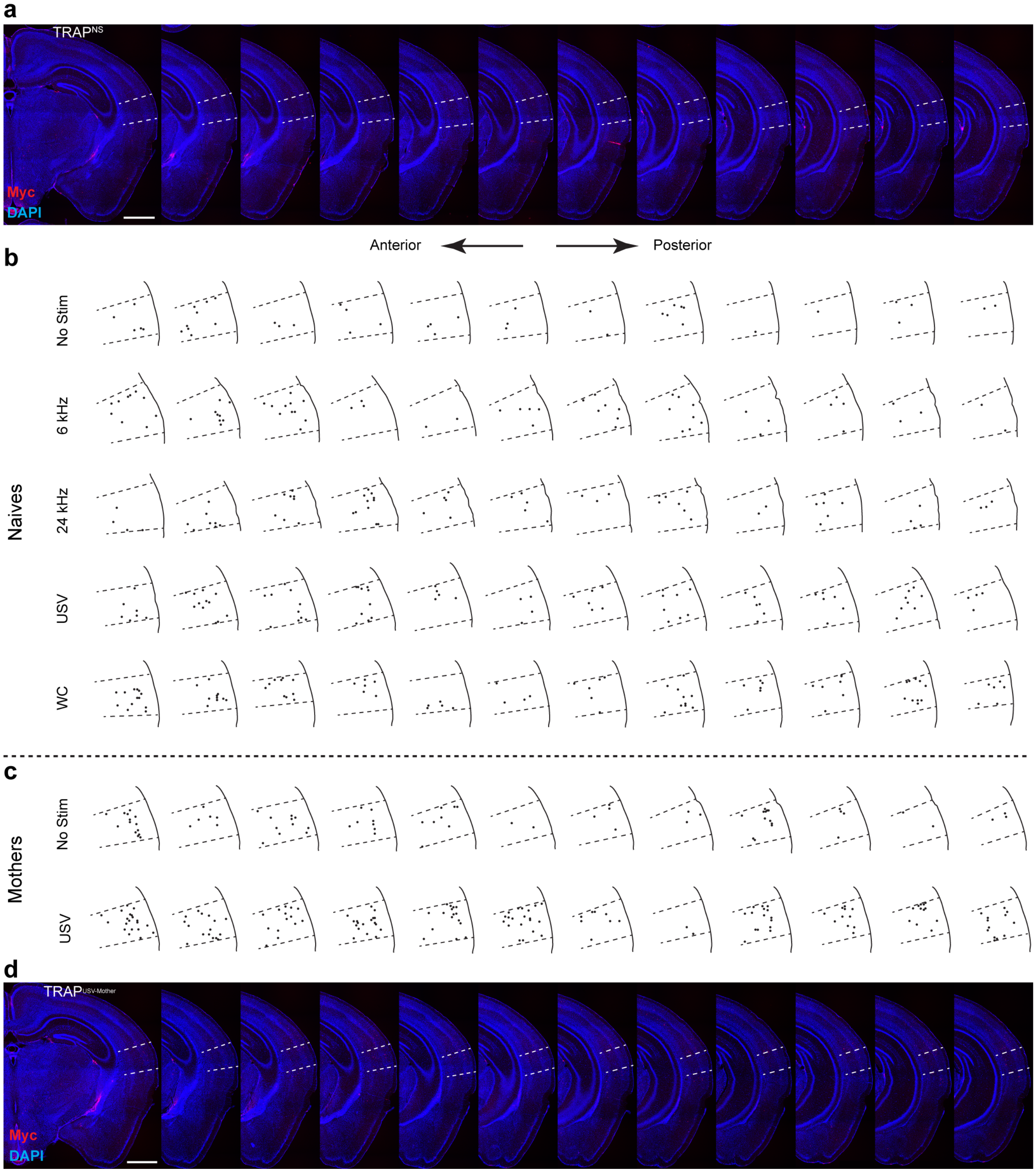
a
TRAP^NS
Myc
DAPI
Anterior
Posterior
b
Naives
No Stim
6 kHz
24 kHz
USV
WC
c
Mothers
No Stim
USV
d
TRAP^USV-Mother
Myc
DAPI

Supplementary Figure 4. TRAP cell counting from sequential brain slices of A1

(a) 12 sequential fluorescent micrographs of coronal brain slices from anterior to posterior positions surrounding A1 in a naïve mouse that was not stimulated with sound (4-OHT dose, 15 mg/kg). The first image is Bregma -2.46mm and slices are 40 μm apart. Scale bar, 1 mm. Blue, DAPI; Magenta, anti-myc staining. Dotted lines denote the estimated borders of A1, which were determined manually based on the DAPI staining only and always blind to the experimental condition. **(b)** Drawings of the exact positions of the neurons (black dots), identified within the borders of A1 in all slices. Top row corresponds to the images in 'a'. Additional rows are representative examples from different experimental groups (one mouse per row representing the following sound stimuli: 6 kHz, 24 kHz, USV, and WC). **(c)** Two representative examples from counting TRAPed neurons in mothers. Top: an example from a mother that was not stimulated with sounds ("No Stim"). Bottom: An example from a mother stimulated with USV. **(d)** The coronal slices from which the data in 'c'- USVwere drawn. Scale bar, 1 mm.

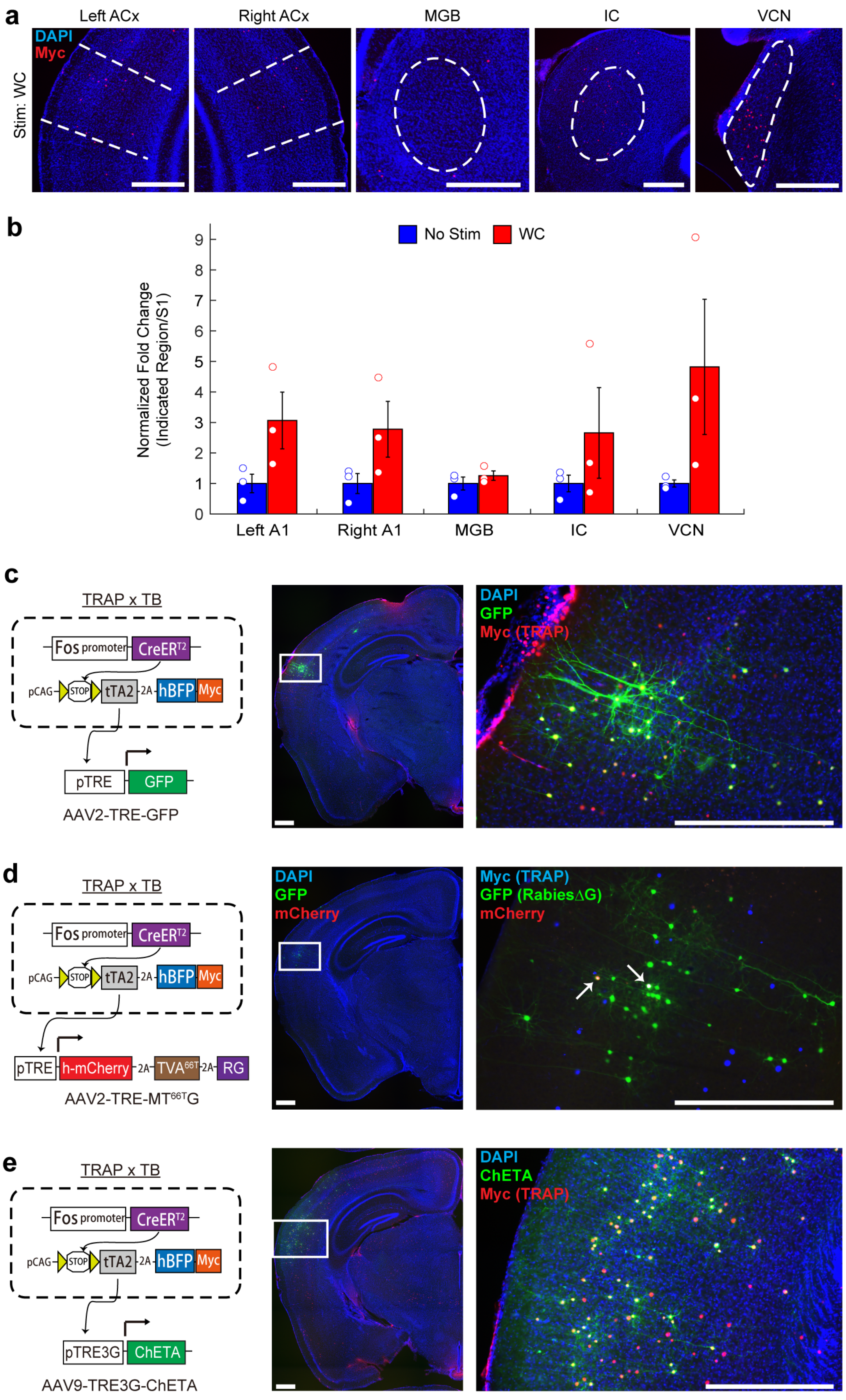
a
Left ACx
Right ACx
MGB
IC
VCN
DAPI
Myc
Stim: WC
b
No Stim
WC
Normalized Fold Change
(Indicated Region/S1)
9
8
7
6
5
4
3
2
1
0
Left A1
Right A1
MGB
IC
VCN
c
TRAP x TB
Fos promoter
CreER T2
pCAG
STOP
tTA2
2A
hBFP
Myc
pTRE
GFP
AAV2-TRE-GFP
DAPI
GFP
Myc (TRAP)
d
TRAP x TB
Fos promoter
CreER T2
pCAG
STOP
tTA2
2A
hBFP
Myc
pTRE
h-mCherry
2A
TVA 66T
2A
RG
AAV2-TRE-MT 66T G
DAPI
GFP
mCherry
Myc (TRAP)
GFP (RabiesΔG)
mCherry
e
TRAP x TB
Fos promoter
CreER T2
pCAG
STOP
tTA2
2A
hBFP
Myc
pTRE3G
ChETA
AAV9-TRE3G-ChETA
DAPI
ChETA
Myc (TRAP)

Supplementary Figure 5. Additional features of the TRAP x TB system: proof of concept

(a) Representative fluorescent micrographs of the left and right primary auditory cortices (Left A1 and Right A1), medial geniculate body in the thalamus (MGB), inferior colliculus in the midbrain (IC), and ventral cochlear nucleus (VCN), all from a single mouse stimulated with WC during TRAPing. All slices are stained for myc (magenta). Scale bars, 500 μm. **(b)** Quantification of the TRAPed cells in five brain regions of the auditory pathway in comparison to S1 (relative density), and normalized to the 'No Stim' condition (mean ± SEM; 'No Stim' N=3 mice, 'WC' N=3 mice). Data presented in the main manuscript is from the right A1. Notably, our calibration of the system with a low dose of 4-OHT was aimed for minimal noise in the auditory cortex. Under these conditions some brain regions still showed robust recombination (e.g., cochlear nucleus), but other regions (e.g., thalamus) showed near-zero recombination and may need higher doses of 4-OHT (as in 'a'). Thus, 4-OHT optimization conditions vary and could be adjusted for different brain regions. **(c-e)** Several possible applications of the TRAP x TB system. **(c)** *AAV-TRE-GFP* was injected to the left A1 of a mouse two weeks prior to injection of 50 mg/kg 4-OHT without sound stimulation (Left: schematic of the genetic design). GFP expression was restricted to TRAPed cells only (note the yellow nucleus). Right photograph is a magnified version taken from the middle micrograph. Scale bars, 500 μm. **(d)** Rabies tracing from TRAPed cells to visualize the local presynaptic landscape of TRAPed cells. Left: schematic of the genetic design. *AAV-TRE-histone2B-mCherry-2A-TVA^{66T}-2A-RG (Rabies Glycoprotein)* was injected to the left A1 of a mouse two weeks prior to injection of 25 mg/kg 4-OHT without sound stimulation. 7 days after injection of 4-OHT, EnvA-pseudotyped rabies virus (deleted its G and replaced to coding GFP sequence; RabiesΔG) was injected to the same place as the injection site of AAV. After 5 days from injection of rabies virus, mice were sacrificed and processed. mCherry expression and the starter cells were restricted to TRAPed cells only (note the white nucleus). White arrows show two starter cells (2 more starter cells were found in other sections in this experiment). Right photograph is a magnified version taken from the middle micrograph. Scale bars, 500 μm. **(e)** *AAV-TRE3G-ChETA-mCherry* was injected to the left A1 of a mouse two weeks prior to injection of 50 mg/kg 4-OHT without sound stimulation (Left: schematic of the genetic design). ChETA expression was largely restricted to TRAPed cells (note the yellow nucleus). Right photograph is a magnified version taken from the middle micrograph. Scale bars, 500 μm.

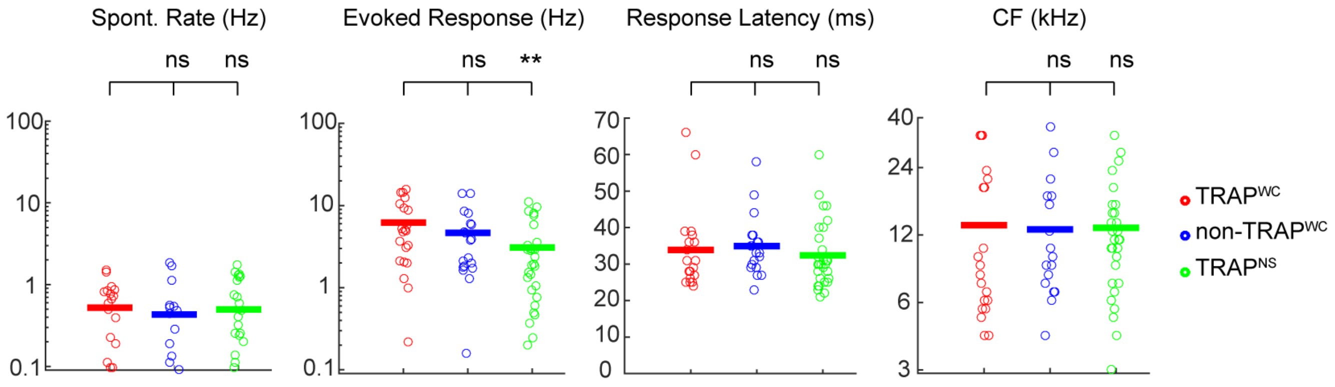

Supplementary Figure 6. Basic response properties to pure tones stimulation are not different between the different groups in WC-stimulated and non-stimulated mice

Comparative analysis of basic response properties of the two neuronal groups in mice TRAPed with WCs ($TRAP^{WC}$ and non-$TRAP^{WC}$) and an additional control group ($TRAP^{NS}$). Spontaneous firing rates ($p=0.55$, Kruskal-Wallis test) and CF ($p=0.63$, Kruskal-Wallis test) were not significantly different. One exception is that $TRAP^{NS}$ had significantly lower evoked rates than $TRAP^{WC}$ (**, $p=0.005$, post hoc Fisher's LSD test after significant Kruskal-Wallis test). This exception is due to a relatively large group of weakly responsive neurons not evident in most other groups.

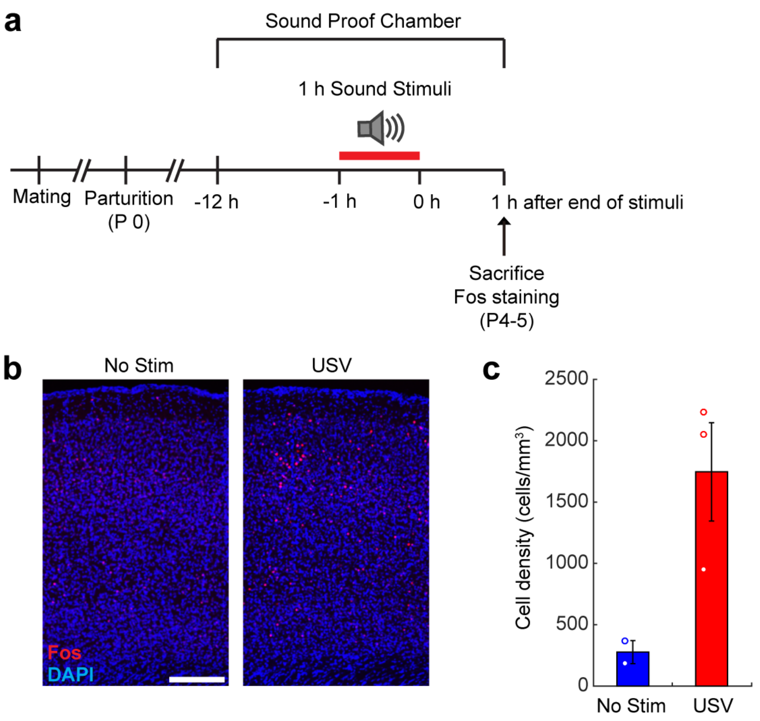

Supplementary Figure 7. Fos expression is lower in mothers compared to naïve virgins

(a) The experimental protocol for Fos staining in mothers. **(b)** Representative fluorescent micrographs of the primary auditory cortices stained with anti-Fos in mothers stimulated with USV or without sound. Scale bar, 200 μm. **(c)** Quantification of the density of Fos positive cells in A1 (mean ± SEM; 'No Stim' N=2 mice, 'USV' N=3 mice). The number of Fos positive cells in both 'No Stim' and 'USV' are lower than in naïve virgins (compare to Supplementary Fig. 3f).

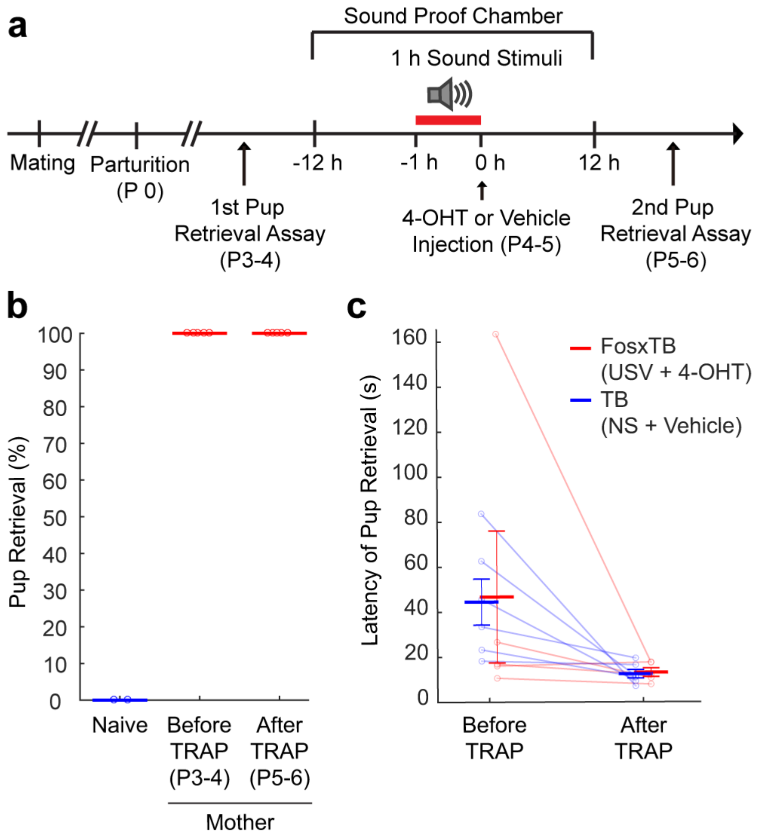

Supplementary Figure 8. Maternal behaviors are not disrupted by TRAPing

(a) The experimental protocol for pup retrieval assays and TRAP in mothers. **(b)** The number of pup retrieved (out of 5) by naïve virgins and in mothers before and after TRAPing. All mothers retrieved all pups while naïves did not retrieve any of the pups (‘Naïve’, N=2 mice, ‘Mother’, N=5 mice). **(c)** Pup retrieval latency of the mothers. Each point is an average of all 5 successful attempts to retrieve the pups. Mothers from *TRAP x TB* or *TB* mice were tested with 25mg/kg 4-OHT or vehicle injection (‘*TRAP x TB*’, N = 5 mothers, ‘*TB*’, N=5 mothers). Note that after the TRAPing procedure, mothers retrieved pups with shorter latency. This normal improvement is not a result of TRAPing but rather a result of their experience and learning. There was no difference between *TRAP x TB* and *TB* mothers. Thus, the heterozygotic *Fos* gene in *TRAP x TB* mice did not affect maternal behaviors.

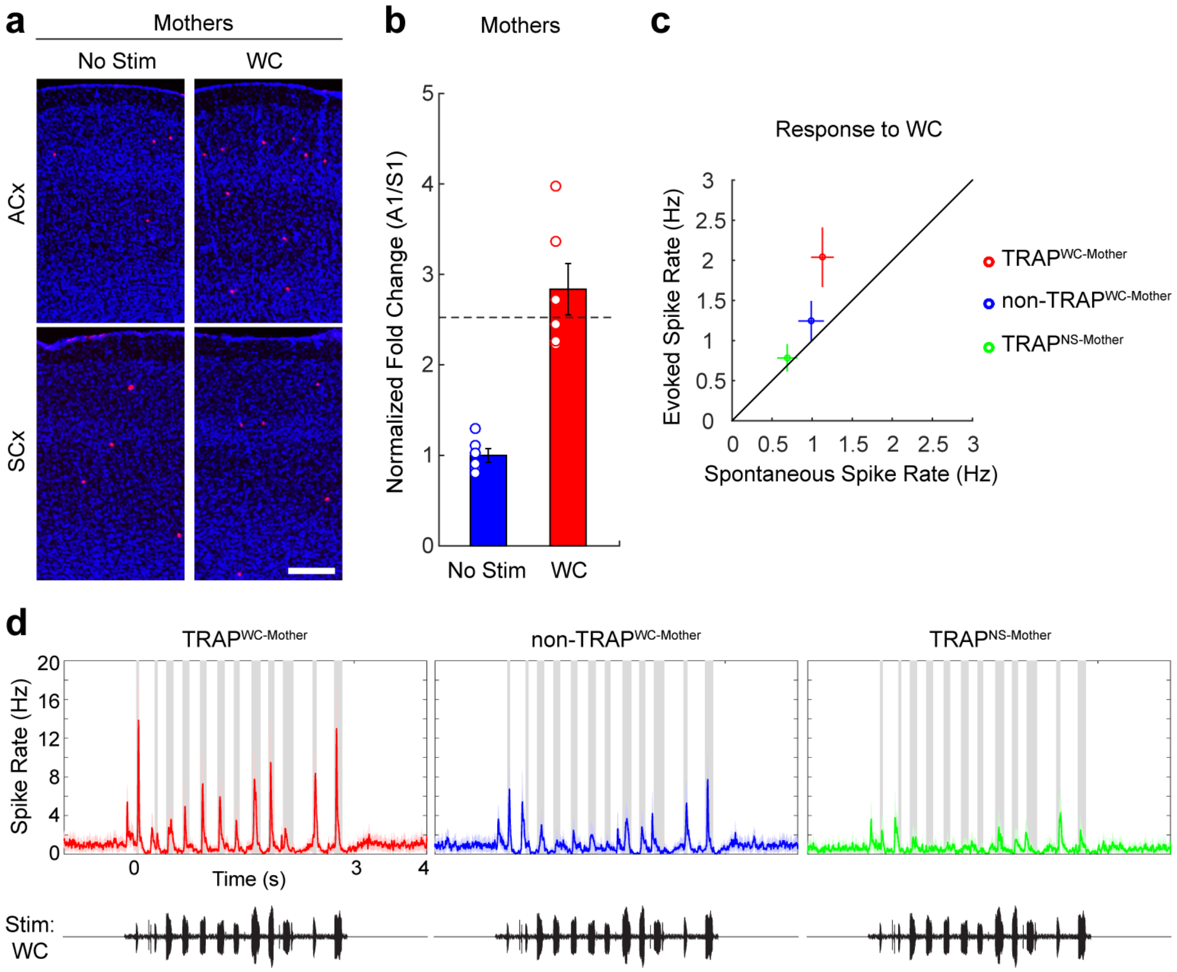

Supplementary Figure 9. TRAPing with WCs induced similar numbers of neurons in A1 of mothers and naïves with no apparant physiological signature

(a) Representative fluorescent micrographs of the A1 (top row) and S1 (bottom row) in *TRAP x TB* mice stimulated with WC or without sound. Each column shows images of ACx and SCx from the same mouse. Scale bar, 200 µm. **(b)** Quantification of the fold induction of TRAPed cells in A1 as compared to S1 (relative density), normalized to the 'No Stim' condition (mean ± SEM; Mothers: 'No Stim' N=6 mice; same plot as in Fig 4c in mothers, 'WC' N=6 mice). WCs induced higher number of the TRAPed cells as compared to the 'No Stim' mother control (***, $p < 0.001$, post hoc Fisher's LSD test after significant Two-way Anova test). However, the efficiency in naives and mothers was similar (dotted line show the induction level in naïve virgins; same as Fig. 1d) **(c)** Plots of evoked vs spontaneous spike rates from all the recorded neurons in all the maternal groups (TRAP$^{\text{WC-Mother}}$; n=35 neurons, N=7 mice, non-TRAP$^{\text{WC-Mother}}$; n=30 neurons, N=7 mice, TRAP$^{\text{NS-Mother}}$; n=21 neurons, N=6 mice). **(d)** Average PSTHs of all neurons in response to WCs from three neuronal groups denoted above. The voltage trace of the WC stimulus is shown beneath the rasters (Compare to Fig. 3b,d). There was no statistical difference between mothers and naives (p=0.53, Two-way Anova test).

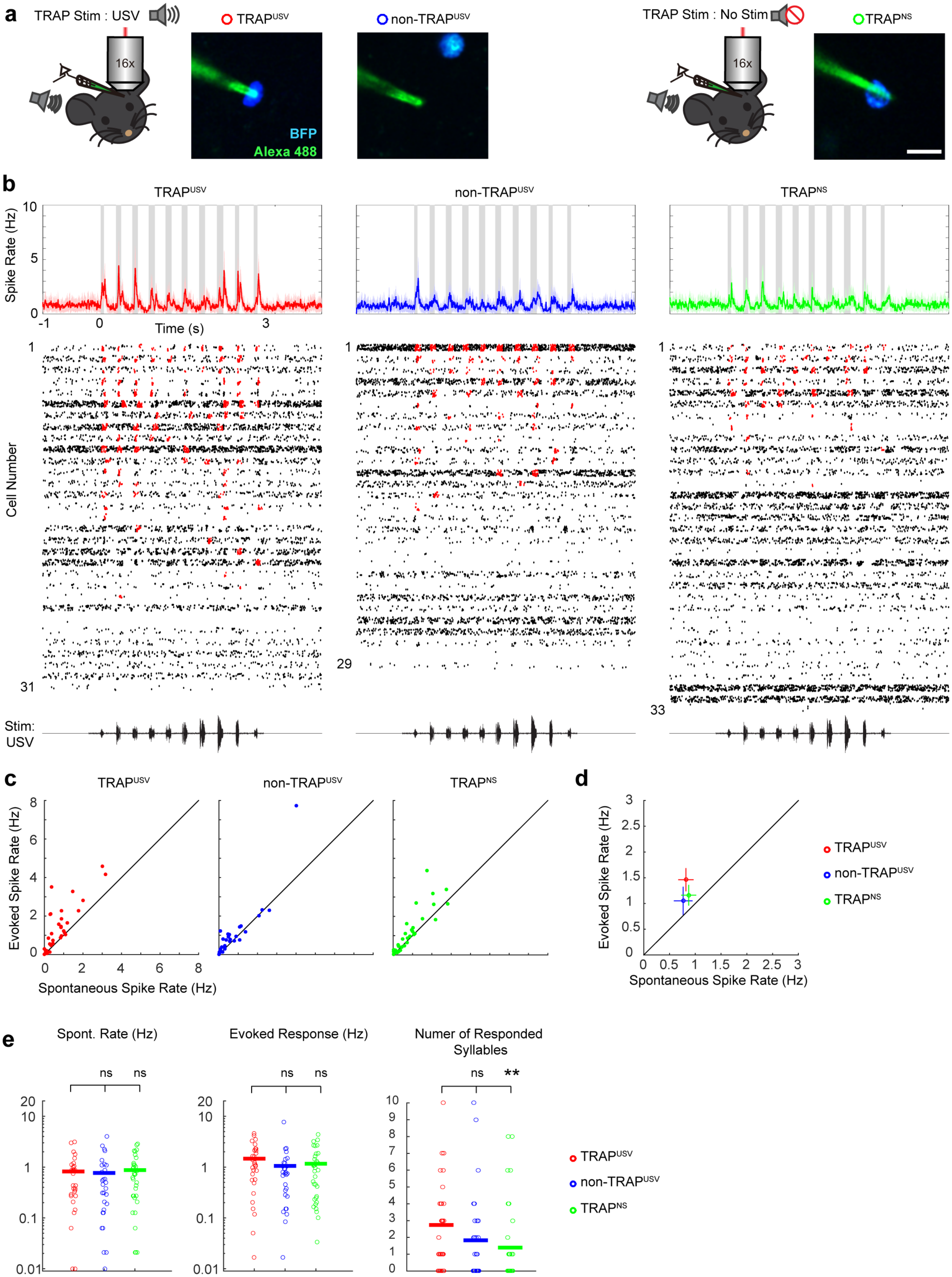

a
TRAP Stim : USV
16x
TRAP^USV
BFP
Alexa 488
non-TRAP^USV
TRAP Stim : No Stim
TRAP^NS
b
TRAP^USV
non-TRAP^USV
TRAP^NS
Spike Rate (Hz)
Time (s)
Cell Number
Stim: USV
c
Evoked Spike Rate (Hz)
Spontaneous Spike Rate (Hz)
d
Evoked Spike Rate (Hz)
Spontaneous Spike Rate (Hz)
e
Spont. Rate (Hz)
Evoked Response (Hz)
Numer of Responded Syllables
ns
ns
**

Supplementary Figure 10. USV-TRAPed cells in naïve females respond slightly, but not significantly, stronger to USVs

(a) Schematic representation of the three neuronal groups from two experimental groups. We recorded from TRAPed and non-TRAPed neurons in mice stimulated with USVs ("USV") and from TRAPed neurons in mice that were not stimulated with sound ("No Stim", green). Scale bar, 10 μm. **(b)** Top: average PSTHs of all neurons in response to USVs from three neuronal groups. Bottom: raster plots of all the recorded neurons ($TRAP^{USV}$; n=31 neurons, N=9 mice, non-$TRAP^{USV}$; n=29 neurons, N=9 mice, $TRAP^{NS}$; n=33 neurons, N=8 mice). Each raster of each neuron is composed of 60 trials. Red lines correspond to spikes that were statistically above the baseline rate around a syllable. The voltage trace of the USV stimulus is shown beneath the rasters. **(c)** Plots of evoked vs spontaneous spike rates from all the recorded neurons in all groups shown in 'b'. Each dot indicates a single neuron. **(d)** Plots of the mean ± SEM evoked vs spontaneous spike rates of the neurons shown in 'b' and 'c'. **(e)** Basic response properties to USVs of all neurons from the three groups. Each circle represents an individual cell. The line is the mean. Spontaneous firing rates (p=0.62, Kruskal-Wallis test) and evoked firing rate (p=0.18, Kruskal-Wallis test) were not significantly different between the groups. One exception is that $TRAP^{NS}$ had significantly lower number of responded syllables than $TRAP^{USV}$ (**, p=0.008, post hoc Fisher's LSD test after significant Kruskal-Wallis test).

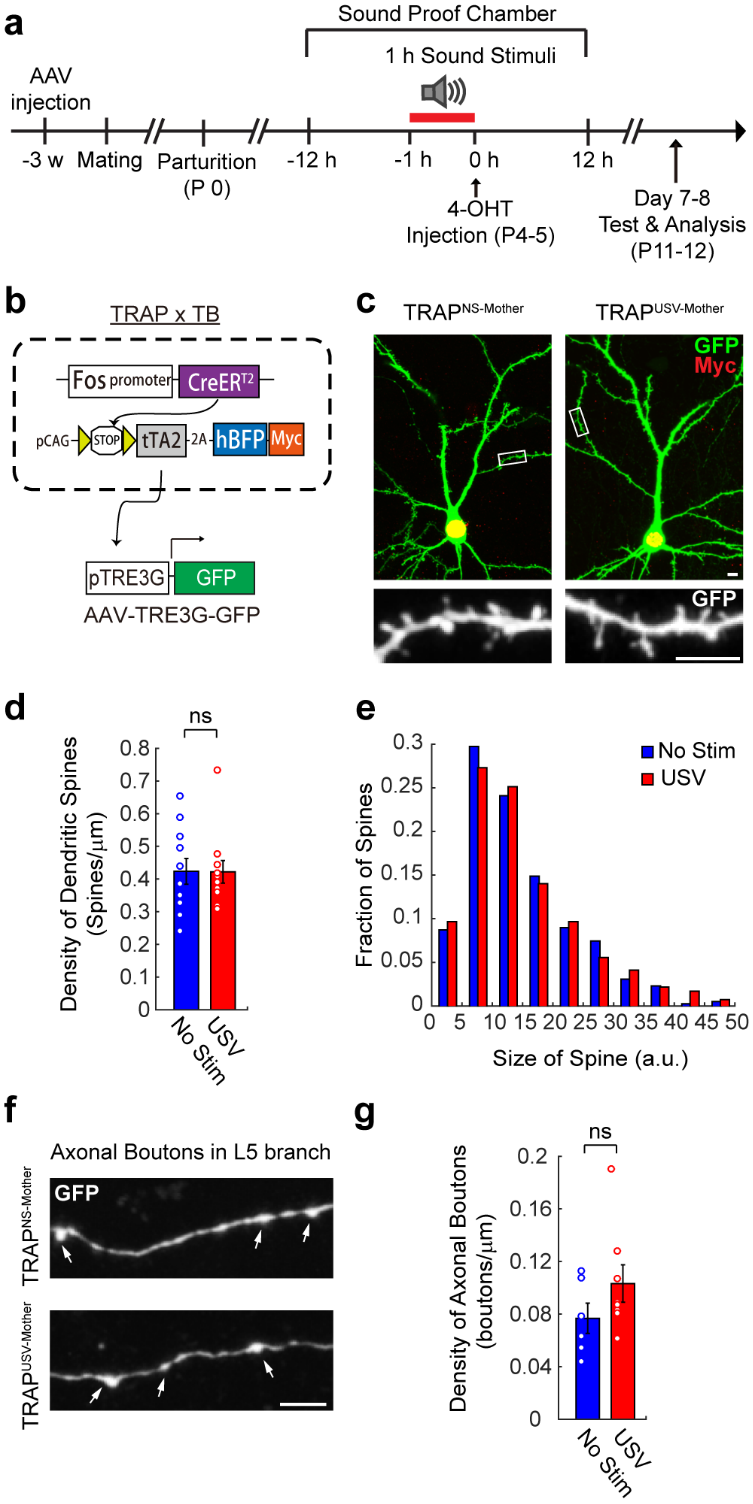

Supplementary Figure 11. USV-TRAPed and non-TRAPed neighbors in mothers have similar densities of dendritic spines and axonal boutons

(a) The experimental protocol for visualization of dendritic spines and axonal boutons in TRAPed cells in mothers. **(b)** A schematic of the genetic components. **(c)** Representative projection images from two different groups injected with *AAV-TRE3G-GFP* to the left A1 of a mouse two weeks prior to injection of 25 mg/kg 4-OHT with or without USV stimulation. The bottom row shows the magnified images from the upper images. Scale bar, 5 µm. **(d)** Quantification of the spine density of apical dendrites in TRAP$^{NS\text{-}Mother}$ (n=11 neurons, N=4 mice) and TRAP$^{USV\text{-}Mother}$ (n=11 neurons, N=5 mice). There was no difference in the spine density between TRAP$^{USV\text{-}Mother}$ and TRAP$^{NS\text{-}Mother}$ (p=0.90, Mann-Whitney U test). **(e)** Quantification of the spine size of apical dendrites in TRAP$^{NS\text{-}Mother}$ (n=390 spines from 10 neurons, N=4 mice) and TRAP$^{USV\text{-}Mother}$ (n=414 spines from 10 neurons, N=5 mice). Spine size values are in arbitrary units (a.u.). There was no difference in the distribution of spine size between two groups (p=0.96, Kolmogorov-Smirnov test). **(f)** Representative confocal projection images of one axonal branch in L5 from a labeled L2/3 neuron from a TRAP$^{USV\text{-}Mother}$ neuron and a TRAP$^{NS\text{-}Mother}$ neuron. *En-passant* boutons bulging from the axon are marked with white arrows. Scale bar, 5 µm. **(g)** Quantification of axonal bouton density of L5 branches in TRAP$^{NS\text{-}Mother}$ (n=6 neurons, N=4 mice) and TRAP$^{USV\text{-}Mother}$ (n=8 neurons, N=5 mice). There was no difference in the density of axonal boutons between the two groups (p=0.23, Mann-Whitney U test).

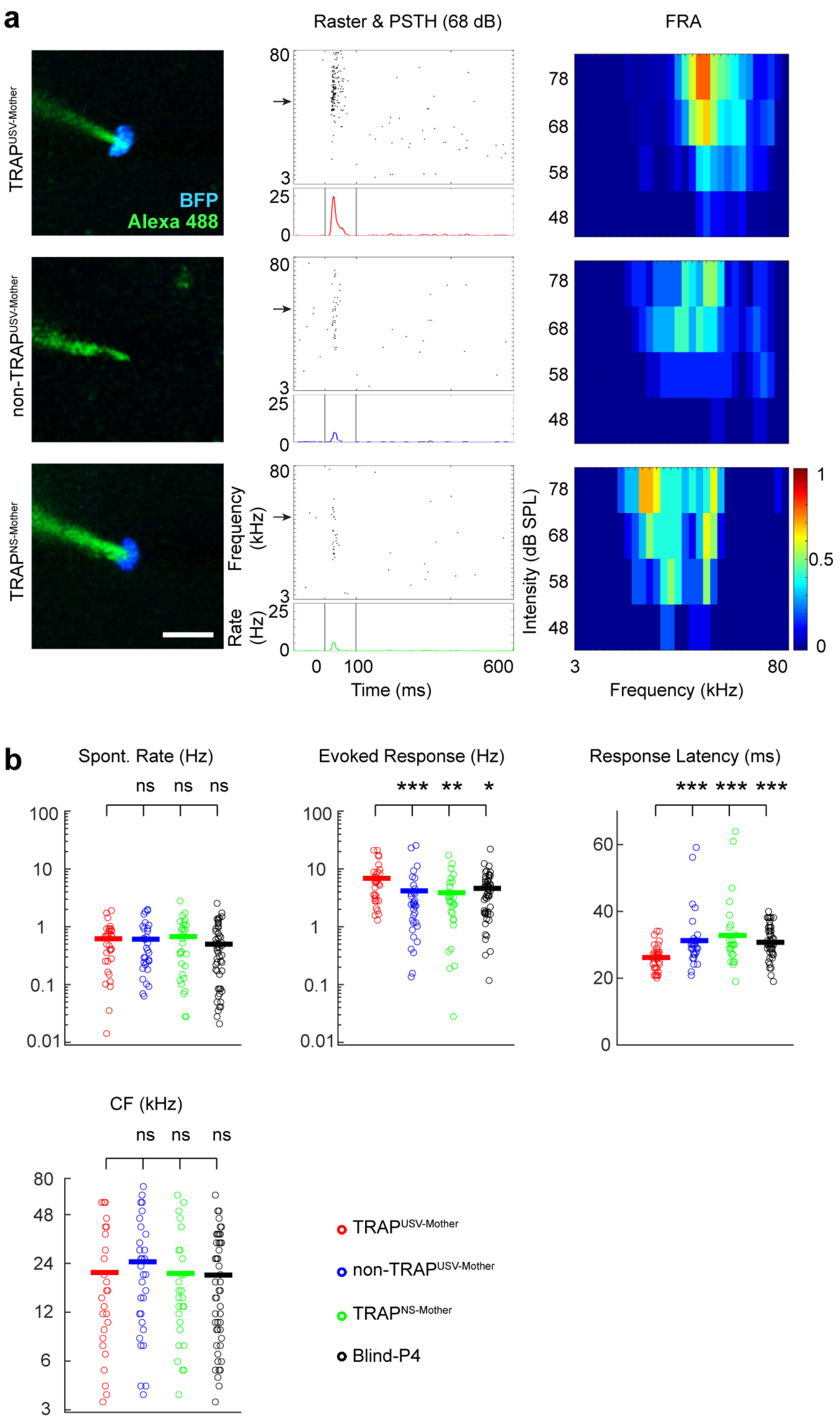

Supplementary Figure 12. Unique properties of USV-TRAPed neurons in mothers: fast and strong responses to pure tones

(a) Representative examples from the four groups of neurons recorded in mothers. Left: two-photon micrograph of the electrode (green) and BFP signal (blue) from each group. Scale bar, 10 µm. Middle and right: Raster plots, PSTHs, and FRAs in response to pure tones. Color bar: normalized firing rates. **(b)** Comparative analysis of basic response properties of the different neuronal groups in mothers. Spontaneous firing rates ($p=0.25$, Kruskal-Wallis test) and CF ($p=0.81$, Kruskal-Wallis test) were not significantly different. TRAPed neurons from $TRAP^{USV\text{-}Mother}$ had significantly higher evoked firing rates and shorter response latency as compared to the controls (**, $p < 0.01$; ***, $p < 0.001$; ns, not significant, post hoc Fisher's LSD test after significant Kruskal-Wallis test)).

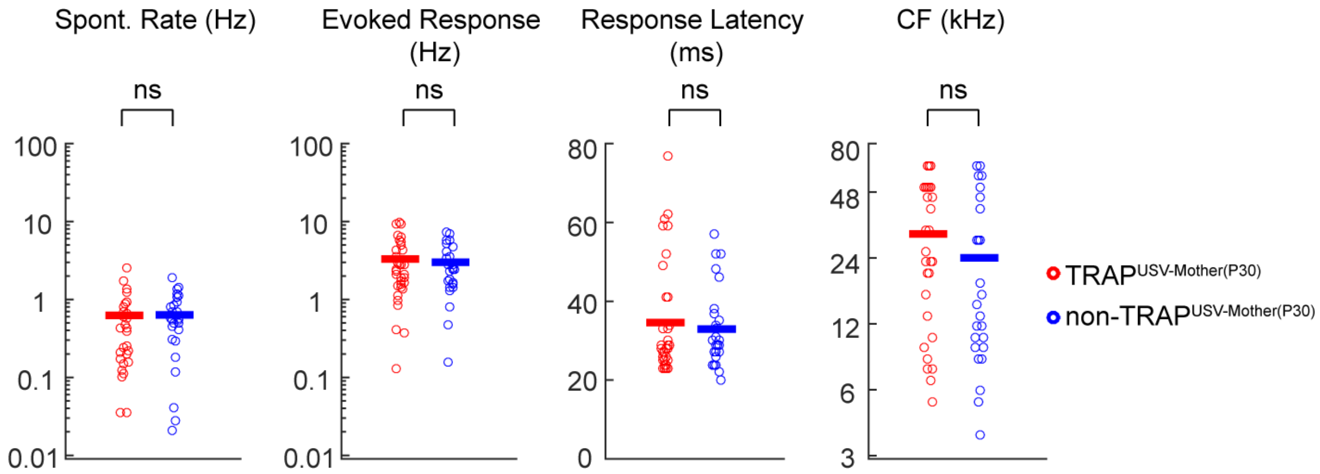

Supplementary Figure 13. USV-TRAPed neurons in mothers following weaning have similarly variable responses to pure tones

Comparative analysis of basic response properties of the neurons in mothers TRAPed with USV at P4 and patched at P30 ($TRAP^{USV\text{-}Mother(P30)}$) and their non-TRAPed neighbors ($non\text{-}TRAP^{USV\text{-}Mother(P30)}$). Spontaneous firing rates (p=0.42, Mann-Whitney U test), Evoked firing rates (p=0.99, Mann-Whitney U test), response latency (p=0.64, Mann-Whitney U test) and CF (p=0.15, Mann-Whitney U test) were not significantly different.

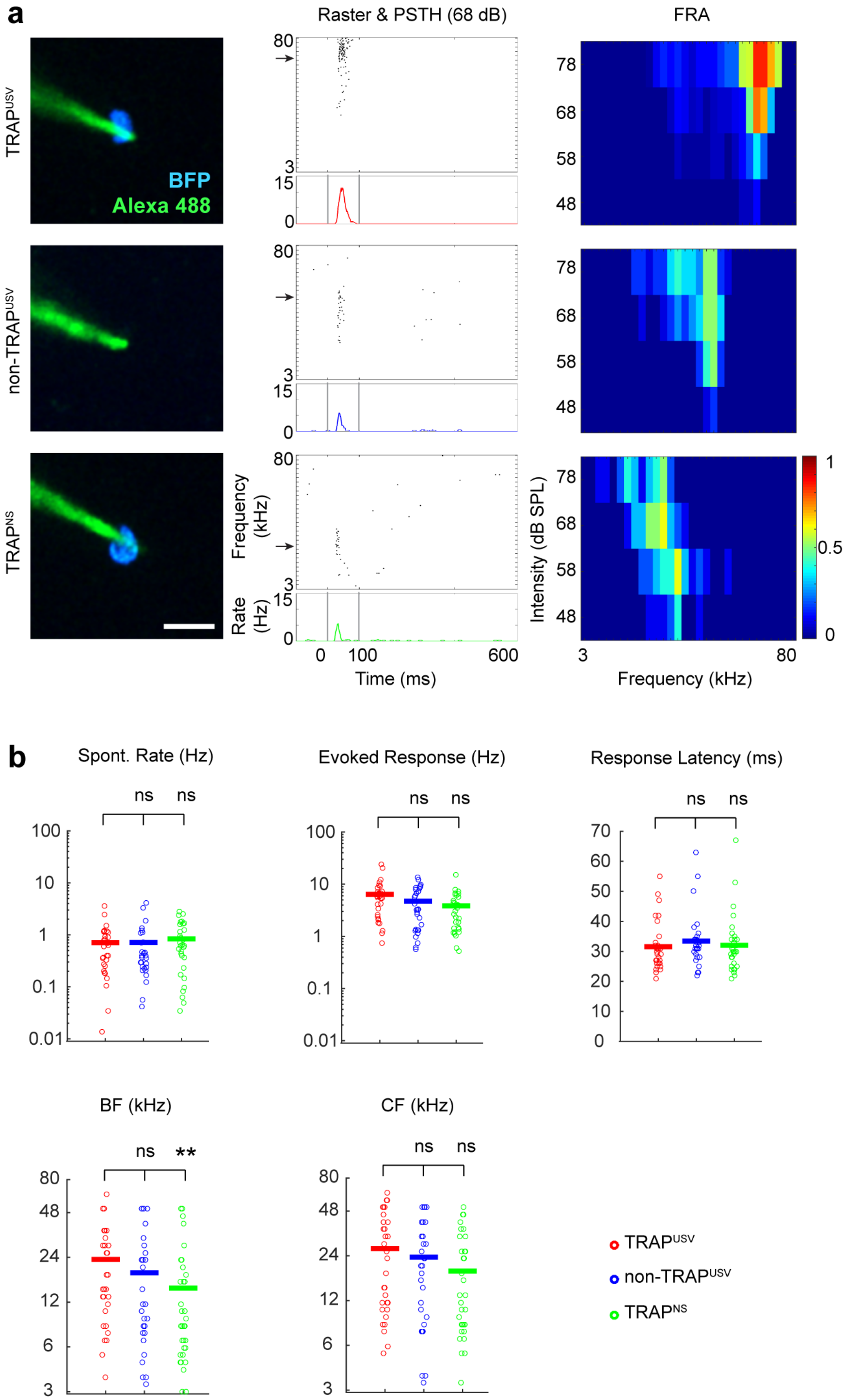

Supplementary Figure 14. USV-TRAPed neurons in naive females have similarly variable responses to pure tones

(a) Representative examples from the three groups of neurons recorded in the two groups of mice. Left: two-photon micrograph. Scale bar, 10 µm. Middle and right: Raster plots, PSTHs, and FRAs in response to pure tones. **(b)** Comparative analysis of basic response properties of the different neuronal groups in naives TRAPed with USV. Spontaneous firing rates (p=0.65, Kruskal-Wallis test), evoked firing rates (p=0.08, Kruskal-Wallis test), latencies (p=0.41, Kruskal-Wallis test), and CF (p=0.15, Kruskal-Wallis test) were similar across groups. One exception is that $TRAP^{NS}$ had lower BF than $TRAP^{USV}$ (**, p=0.008, post hoc Fisher's LSD test after significant Kruskal-Wallis test).

Supplementary Table 1. Physiological properties of TRAPed and non-TRAPed cells in response to pure tones across the dataset

Exp. Group	Animals (N)	Cells (n)	Depth (μm)	Spontaneous spike rate (Hz)	Evoked response (Hz)	Response latency (ms)	BF (kHz)	CF (kHz)	Shown in
$TRAP^{WC}$	6	21	275 ± 45.9	0.52 ± 0.45	6.22 ± 4.84	33.9 ± 10.9	12.9 ± 10.6	13.3 ± 10.3	Supplementary Figure 6 (red)
$non\text{-}TRAP^{WC}$	6	19	259 ± 48.7	0.43 ± 0.55	4.66 ± 4.06	34.9 ± 8.26	10.1 ± 7.24	12.7 ± 8.48	Supplementary Figure 6 (blue)
$TRAP^{NS}$	8	29	270 ± 51.8	0.50 ± 0.54	3.09 ± 3.05	32.4 ± 9.21	9.51 ± 7.10	12.9 ± 7.28	Supplementary Figure 6(green)
$TRAP^{USV}$	9	31	260 ± 34.3	0.71 ± 0.76	6.36 ± 5.17	31.6 ± 8.09	23.2 ± 15.9	26.8 ± 18.2	Supplementary Figure 14 (red)
$non\text{-}TRAP^{USV}$	9	29	263 ± 36.0	0.71 ± 0.94	4.71 ± 3.78	33.4 ± 9.00	18.9 ± 15.3	23.5 ± 15.8	Supplementary Figure 14 (blue)
$TRAP^{NS}$	8	33	288 ± 52.0	0.84 ± 0.79	3.85 ± 3.01	32.0 ± 9.19	14.9 ± 13.8	18.9 ± 13.9	Supplementary Figure 14 (green)
$TRAP^{USV\text{-}Mother}$	10	33	278 ± 43.0	0.62 ± 0.46	6.88 ± 5.26	26.2 ± 3.75	20.9 ± 16.5	21.1 ± 16.3	Supplementary Figure 12 (red)
$non\text{-}TRAP^{USV\text{-}Mother}$	10	33	270 ± 46.7	0.61 ± 0.57	4.17 ± 5.76	31.2 ± 8.14	20.1 ± 15.2	24.6 ± 18.8	Supplementary Figure 12 (blue)
$TRAP^{NS\text{-}Mother}$	7	29	282 ± 62.0	0.67 ± 0.65	3.88 ± 3.97	32.8 ± 10.0	15.3 ± 13.4	20.8 ± 16.1	Supplementary Figure 12 (green)
Blind-P4	7	58	287 ± 47.0	0.50 ± 0.53	4.61 ± 3.68	30.7 ± 4.80	17.4 ± 16.9	20.3 ± 15.3	Supplementary Figure 12 (black)
$TRAP^{USV\text{-}Mother(P30)}$	6	34	292 ± 58.2	0.63 ± 0.68	3.30 ± 2.59	34.6 ± 14.3	34.1 ± 21.2	30.9 ± 19.7	Supplementary Figure 13 (red)
$non\text{-}TRAP^{USV\text{-}Mother(P30)}$	6	27	284 ± 49.7	0.63 ± 0.48	3.00 ± 1.91	32.9 ± 9.86	24.4 ± 21.5	24.1 ± 19.7	Supplementary Figure 13 (blue)
$TRAP^{WC\text{-}Mother}$	7	35	279 ± 45.9	0.73 ± 0.54	6.67 ± 5.84	31.5 ± 10.3	10.7 ± 8.95	13.2 ± 10.9	N/A
$non\text{-}TRAP^{WC\text{-}Mother}$	7	30	268 ± 37.2	0.79 ± 0.85	4.38 ± 3.97	31.2 ± 6.72	11.8 ± 8.90	15.7 ± 11.6	N/A
$TRAP^{NS\text{-}Mother}$	6	21	280 ± 68.7	0.91 ± 1.27	4.61 ± 4.28	33.6 ± 9.08	17.1 ± 11.8	17.1 ± 10.4	N/A

All values are mean ± SD.

Supplementary Table 2. Physiological properties of TRAPed and non-TRAPed cells in response to WCs (Wriggling Calls)

Exp. Group	Animals (N)	Cells (n)	Depth (μm)	Spontaneous spike rate (Hz)	Evoked response (Hz)	Number of responded syllables	Shown in
$TRAP^{WC}$	9	33	257 ± 49.4	0.82 ± 0.69	1.80 ± 2.33	4.79 ± 2.62	Figure3 (red)
$non\text{-}TRAP^{WC}$	9	28	249 ± 46.8	0.74 ± 0.72	0.99 ± 1.37	2.89 ± 2.00	Figure 3 (blue)
$TRAP^{NS}$	8	29	270 ± 51.8	0.61 ± 0.68	0.88 ± 1.41	2.59 ± 2.29	Figure 3 (green)
$TRAP^{WC\text{-}Mother}$	7	35	279 ± 45.9	1.01 ± 0.75	2.04 ± 2.97	4.34 ± 3.62	Supplementary Figure 9 (red)
$non\text{-}TRAP^{WC\text{-}Mother}$	7	30	268 ± 37.2	0.91 ± 0.83	1.24 ± 1.78	2.93 ± 2.91	Supplementary Figure 9 (blue)
$TRAP^{NS\text{-}Mother}$	6	21	280 ± 68.7	0.60 ± 0.48	1.28 ± 1.54	2.24 ± 2.57	Supplementary Figure 9 (green)

All values are mean ± SD.

Supplementary Table 3. Statistical analysis in response to WCs

	Spontaneous spike rate (Hz)		Evoked response (Hz)		Number of responded syllables	
Statistical test	Chi-sq value	P value	Chi-sq value	P value	Chi-sq value	P value
Kruskal-Wallis	2.46	0.292	10.82	0.0045	13.05	0.0015
Fisher's LSD test $TRAP^{WC}$ vs non-$TRAP^{WC}$		N/A		0.0284		0.0067
Fisher's LSD test $TRAP^{WC}$ vs $TRAP^{NS}$		N/A		0.0014		0.0007
Fisher's LSD test non-$TRAP^{WC}$ vs $TRAP^{NS}$		N/A		0.3485		0.5422
Degree of Freedom	2		2		2	
Shown in	Figure 3e		Figure 3e		Figure 3e	

Supplementary Table 4. Statistical analysis in response to pure tones

	Spontaneous spike rate (Hz)		Evoked response (Hz)		Response latency (ms)		BF (kHz)		CF (kHz)	
Statistical test	Chi-sq value	P value	Chi-sq value	P value	Chi-sq value	P value	Chi-sq value	P value	Chi-sq value	P value
Kruskal-Wallis	1.2	0.5495	7.98	0.0185	1.96	0.3745	0.24	0.8861	0.92	0.6319
Fisher's LSD test TRAP^{WC} vs non-TRAP^{WC}		N/A		0.2717		N/A		N/A		N/A
Fisher's LSD test TRAP^{WC} vs TRAP^{NS}		N/A		0.0052		N/A		N/A		N/A
Fisher's LSD test non-TRAP^{WC} vs TRAP^{NS}		N/A		0.1259		N/A		N/A		N/A
Degree of Freedom	2		2		2		2		2	
Shown in	Supplementary Figure 6		Supplementary Figure 6		Supplementary Figure 6		Figure 3f		Supplementary Figure 6	
Statistical test	Chi-sq value	P value	Chi-sq value	P value	Chi-sq value	P value	Chi-sq value	P value	Chi-sq value	P value
Kruskal-Wallis	4.09	0.2522	14.89	0.0019	22.31	0.000056	4.36	0.2253	0.95	0.8132
Fisher's LSD test TRAP$^{USV-Mother}$ vs non-TRAP$^{USV-Mother}$		N/A		0.0004		0.0009		N/A		N/A
Fisher's LSD test TRAP$^{USV-Mother}$ vs TRAP$^{NS-Mother}$		N/A		0.0028		0.0002		N/A		N/A
Fisher's LSD test TRAP$^{USV-Mother}$ vs Blind-P4		N/A		0.0439		0.000009		N/A		N/A
Fisher's LSD test non-TRAP$^{USV-Mother}$ vs TRAP$^{NS-Mother}$		N/A		0.6796		0.6254		N/A		N/A
Fisher's LSD test non-TRAP$^{USV-Mother}$ vs Blind-P4		N/A		0.0500		0.4864		N/A		N/A
Fisher's LSD test TRAP$^{NS-Mother}$ vs Blind-P4		N/A		0.1564		0.9037		N/A		N/A
Degree of Freedom	3		3		3		3		3	
Shown in	Supplementary Figure 12		Supplementary Figure 12		Supplementary Figure 12		Figure 5d		Supplementary Figure 12	
Statistical test	P value		P value		P value		P value		P value	
Mann-Whitney U test TRAP$^{USV-Mother(P30)}$ vs non-TRAP$^{USV-Mother(P30)}$	0.4203		0.9942		0.6414		0.0381		0.1539	
Shown in	Supplementary Figure 13		Supplementary Figure 13		Supplementary Figure 13		Figure 6d		Supplementary Figure 13	
Statistical test	Chi-sq value	P value	Chi-sq value	P value	Chi-sq value	P value	Chi-sq value	P value	Chi-sq value	P value
Kruskal-Wallis	0.88	0.6456	4.97	0.0831	1.77	0.4136	7	0.0303	3.82	0.1477
Fisher's LSD test TRAP^{USV} vs non-TRAP^{USV}		N/A		N/A		N/A		0.1796		N/A
Fisher's LSD test TRAP^{USV} vs TRAP^{NS}		N/A		N/A		N/A		0.0082		N/A

Fisher's LSD test non-TRAP^{USV} vs TRAP^{NS}		N/A		N/A		N/A		0.2161		N/A
Degree of Freedom	2		2		2		2		2	
Shown in	Supplementary Figure 14		Supplementary Figure 14		Supplementary Figure 14		Supplementary Figure 14		Supplementary Figure 14	

Supplementary Table 5. Physiological properties of TRAPed cells with pure tones in response to pure tones

Exp. Group	Animals (N)	Cells (n)	Depth (μm)	Spontaneous spike rate (Hz)	Evoked response (Hz)	Response latency (ms)	BF (kHz)	CF (kHz)	Shown in
$TRAP^{6kHz}$	4	14	229 ± 69.8	0.72 ± 1.10	3.63 ± 3.56	30.0 ± 7.31	13.7 ± 11.6	11.2 ± 10.3	N/A
$TRAP^{24kHz}$	5	17	235 ± 43.1	0.71 ± 0.90	5.94 ± 5.61	31.2 ± 12.9	20.9 ± 12.5	21.2 ± 11.9	N/A
$TRAP^{NS}$	3	21	241 ± 48.3	0.47 ± 0.61	3.33 ± 3.02	30.0 ± 7.37	8.63 ± 4.81	14.3 ± 7.02	N/A

All values are mean ± SD.

Supplementary Table 6. Physiological properties of TRAPed and non-TRAPed cells in response to USVs (Ultrasonic Vocalizations)

Exp. Group	Animals (N)	Cells (n)	Depth (μm)	Spontaneous spike rate (Hz)	Evoked response (Hz)	Number of responded syllables	Shown in
$TRAP^{USV}$	9	31	260 ± 34.3	0.82 ± 0.79	1.46± 1.57	2.74 ± 2.72	Supplementary Figure 10 (red)
$non\text{-}TRAP^{USV}$	9	29	263 ± 36.0	0.77 ± 0.93	1.05 ± 1.65	1.83 ± 2.66	Supplementary Figure 10 (blue)
$TRAP^{NS}$	8	33	288 ± 52.0	0.87 ± 0.79	1.16 ± 1.34	1.39 ± 2.41	Supplementary Figure 10 (green)
$TRAP^{USV\text{-}Mother}$	10	33	278 ± 43.0	0.89 ± 0.59	2.08 ± 3.20	2.48 ± 2.83	Figure 5(red)
$non\text{-}TRAP^{USV\text{-}Mother}$	10	33	270 ± 46.7	0.74 ± 0.69	1.11 ± 1.29	1.85 ± 2.56	Figure 5 (blue)
$TRAP^{NS\text{-}Mother}$	7	29	282 ± 62.0	0.77 ± 0.69	1.04 ± 1.11	1.38 ± 1.95	Figure 5 (green)
Blind-P4	7	58	287 ± 47.0	0.68 ± 0.74	1.13 ± 1.53	2.64 ± 2.78	Figure 5 (black)
$TRAP^{USV\text{-}Mother(P30)}$	6	34	292 ± 58.2	0.73 ± 0.61	1.18 ± 1.41	2.53 ± 2.49	Figure 6b-e (blue)
$non\text{-}TRAP^{USV\text{-}Mother(P30)}$	6	27	284 ± 49.7	0.70 ± 0.52	0.98 ± 1.09	2.37 ± 2.48	Figure 6b-e (green)

All values are mean ± SD.

Supplementary Table 7. Statistical analysis in response to USVs

	Spontaneous spike rate (Hz)		Evoked response (Hz)		Number of responded syllables	
Statistical test	Chi-sq value	P value	Chi-sq value	P value	Chi-sq value	P value
Kruskal-Wallis	4.9	0.1793	8.49	0.0369	5.78	0.1229
Fisher's LSD test TRAP$^{USV-Mother}$ vs non-TRAP$^{USV-Mother}$		N/A		0.0323		N/A
Fisher's LSD test TRAP$^{USV-Mother}$ vs TRAP$^{NS-Mother}$		N/A		0.0317		N/A
Fisher's LSD test TRAP$^{USV-Mother}$ vs Blind-P4		N/A		0.0057		N/A
Fisher's LSD test non-TRAP$^{USV-Mother}$ vs TRAP$^{NS-Mother}$		N/A		0.9380		N/A
Fisher's LSD test non-TRAP$^{USV-Mother}$ vs Blind-P4		N/A		0.7301		N/A
Fisher's LSD test TRAP$^{NS-Mother}$ vs Blind-P4		N/A		0.8074		N/A
Degree of Freedom	3		3		3	
Shown in	Figure 5c		Figure 5c		Figure 5c	
Statistical test	Chi-sq value	P value	Chi-sq value	P value	Chi-sq value	P value
Kruskal-Wallis	0.96	0.6187	3.39	0.1836	7.27	0.0263
Fisher's LSD test TRAP^{USV} vs non-TRAP^{USV}		N/A		N/A		0.0878
Fisher's LSD test TRAP^{USV} vs TRAP^{NS}		N/A		N/A		0.0078
Fisher's LSD test non-TRAP^{USV} vs TRAP^{NS}		N/A		N/A		0.3773
Degree of Freedom	2		2		2	
Shown in	Supplementary Figure 10		Supplementary Figure 10		Supplementary Figure 10	
Statistical test	P value		P value		P value	
Mann-Whitney U test TRAP$^{USV-Mother(P30)}$ vs non-TRAP$^{USV-Mother(P30)}$	0.9595		0.3234		0.7622	
Shown in	Figure 6c		Figure 6c		Figure 6c	

Supplementary Reference

1. Madisen, L. *et al.* A robust and high-throughput Cre reporting and characterization system for the whole mouse brain. *Nat. Neurosci.* **13**, 133-140 (2010).
